# Rydberg states of alkali atoms on superfluid helium nanodroplets: inside or outside?†
†Electronic supplementary information (ESI) available: Several figures detailing the results of this work. The accuracy of the Chebyshev method is addressed and additional figures are presented for other alkali metal atoms besides Rb. See DOI: 10.1039/c7cp02332d


**DOI:** 10.1039/c7cp02332d

**Published:** 2017-05-16

**Authors:** Johann V. Pototschnig, Florian Lackner, Andreas W. Hauser, Wolfgang E. Ernst

**Affiliations:** a Institute of Experimental Physics , Graz University of Technology , Petersgasse 16 , A-8010 Graz , Austria . Email: andreas.w.hauser@gmail.com ; Email: wolfgang.ernst@tugraz.at

## Abstract

Electronic excitations of an electron bound to an alkali metal ion inside a droplet of superfluid ^4^He are computed *via* a combination of helium density functional theory and the numerical integration of the Schrödinger equation for a single electron in a modified, He density dependent atomic pseudopotential. The application of a spectral method to the radial part of the valence electron wavefunction allows the computation of highly excited Rydberg states. For low principal quantum numbers, the energy required to push the electron outward is larger than the solvation energy of the ion. However, for higher principal quantum numbers the situation is reversed, which suggests the stability of a system where the ion sits inside the droplet while the valence electron orbits the nanodroplet.

## Abstract 2

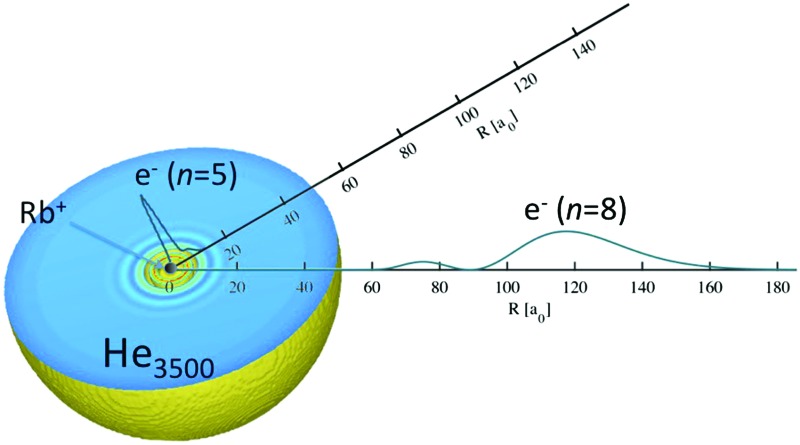
Electronic excitations of an electron bound to an alkali metal ion inside a droplet of superfluid ^4^He are computed *via* a combination of helium density functional theory and the numerical integration of the Schrödinger equation for a single electron in a modified, He density dependent atomic pseudopotential.

## 1 Introduction

In 1999, Platzman and Dykman suggested the placement of electrons on liquid helium as a method to realize quantum bits with long coherence times.^1^ A layer of superfluid helium effectively decouples these qubits from the environment,^2^–^5^ while interbit couplings are realized by electric dipole–dipole interactions. Coherence times in these systems are expected to be in the range of ms if the liquid helium is kept at a temperature of a few mK.^2^,^6^ Obviously, a weakly bound electron floating on a helium surface has to be pinned or spatially confined before it can be addressed, which is typically achieved *via* electromagnetic fields or microelectrodes under the helium layer.

In this article, we would like to link this fundamental problem of quantum information processing with the concept of Rydberg excitation, a well-studied classical field of atomic spectroscopy. In principle, the setup of a highly excited and therefore almost classically localized electron in the field of a nucleus is not too different from the situation of an electron floating on a layer of helium but being pinned to an underlying charge. This article is dedicated to the theoretical study of a hybrid system which lies exactly in between these two situations:^7^,^8^ an ion without the valence electron is submerged in a superfluid ^4^He nanodroplet and the electron takes a position outside this helium shell. Alkali metal atoms were selected to study this system in detail for two reasons. Firstly, they have only one valence electron outside the closed shells, which simplifies the theoretical approach and makes it possible to describe the remaining electrons efficiently with pseudopotentials. Secondly, alkali metal atoms are known to reside on the surface of droplets after adsorption and their excited states have been investigated widely on superfluid helium nanodroplets. It has been shown that they can be excited to higher electronic states without getting detached.^9^–^11^ The experimental findings led to the assumption that for sufficiently high excitations, *i.e.* excitations to Rydberg levels, the remaining ion should sink into the helium droplet while the electron keeps orbiting outside. Golov and Sekatskii^12^,^13^ have been working on a very similar system, in which an electron is orbiting an ionized helium cluster. However, in the case of a Rydberg excitation of a neutral atom adsorbed onto a large droplet of superfluid helium, different mechanisms have to be taken into consideration. Before going into the details of our approach to this fascinating system, a brief overview of previous studies on the electron–helium interaction and the electronic excitation of atoms on helium nanodroplets shall be given.

The interaction between an electron and superfluid helium has been a research topic for several decades. Early works considered Rydberg states of an electron on a planar surface of liquid helium^14^,^15^ and of an electron bound to an ionized helium cluster.^12^,^13^ Bound states of electrons attached to superfluid He clusters^16^ and floating on a superfluid layer^5^,^17^,^18^ have also been studied. Inside of bulk helium, electrons^19^ or negative ions^20^ are known to form local cavities or ‘bubbles’. The spectra and dynamics of electron bubbles have been analyzed by theory^21^–^23^ and experiment.^24^–^26^ In contrast, positive ions are surrounded by high densities of frozen helium, referred to as ‘snowballs’.^27^ Optimized geometries for ions surrounded by He were calculated by *ab initio* methods^28^ or combined approaches^29^,^30^ taking quantum effects into consideration as well. Other authors investigated the solvation of alkali metal ions in superfluid helium droplets with variational,^31^–^33^ diffusion^34^ or path integral Monte Carlo methods.^35^,^36^ Even the dynamics of solvation^37^ and the mobility of ions^38^ in helium droplets have been studied *via* density functional theory. These systems have also been investigated experimentally.^11^,^27^,^39^,^40^,^41^ For alkali metal atoms and their oligomers, numerous experimental studies have been performed by researchers in the field of helium nanodroplet spectroscopy.^9^,^42^–^44^ The lower excited states of atomic Li,^45^ Na,^46^ K,^47^ Rb,^47^–^49^ and Cs^11^,^47^ were investigated. In the case of Li,^45^ Na,^50^–^52^ K,^51^,^52^ and Rb^53^–^55^ exciplexes, complexes consisting of an excited alkali atom and helium atoms were observed upon excitation. Rydberg series were studied for Li,^56^ Na,^57^,^58^ Rb,^59^,^60^ and Cs.^60^,^61^ Besides alkali atoms, the Rydberg states of He in superfluid He nanodroplets have also been investigated.^62^

This article is structured as follows. In Section 2 we discuss our computational approach in detail. The Schrödinger equation for the valence electron of an alkali metal atom is solved in a potential consisting of the standard pseudopotential terms for the unperturbed atom and a repulsive correction which emulates the presence of a spherical helium shell between the ionic core and the valence electron. This allows us to simulate the situation of having the electron orbiting around a helium nanodroplet with an alkali metal ion inside. In Section 3 we test the accuracy of our computational approach to the evaluation of excited states for the valence electron, calculate the solvation energies for alkali metal ions immersed in He droplets of various sizes and analyze the impact of the surrounding helium on these ‘modified’ Rydberg states for a direct comparison to the experimental studies of alkali-metal-doped helium nanodroplets. We discuss the shifts in the positions of electronically excited states and give estimates for principal quantum numbers where sinking of the remaining alkali metal ions into the droplet should occur.

## 2 Methods

We aim to describe the situation of having the ionic core of an alkali metal atom completely immersed in a helium nanodroplet consisting of 128 to 10 000 He atoms, while its single valence electron is orbiting outside the helium droplet. First, the approach for the computation of electronically excited states for the unperturbed alkali metal is presented. We then introduce an additional, repulsive potential term which emulates the presence of a radially symmetric helium layer around the ionic core. This potential can be derived from helium density functional theory calculations, and is combined with the free-atom pseudopotentials in order to provide the perturbed electronic eigenstates and energies.

### 2.1 A numerical solution for the valence-electron wavefunction

The attractive (long range) potential of the singly charged ionic core is described by a set of exponential pseudopotentials taken from ref. 65, which were chosen over those of Fuentealba *et al.*^66^,^67^ or Marinescu *et al.*^68^ due to their considerably better performance for Rydberg states. In atomic units, the pseudopotentials are of the form1
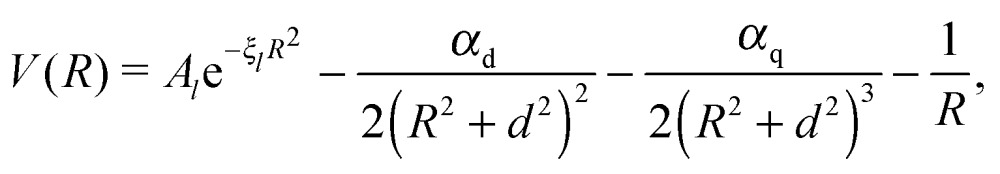
 with *R* denoting the distance between the electron and the ionic core and *d* as a small constant added to the denominator of higher terms in the series expansion to avoid their divergence at the origin. The different contributions modify the 1/*R* potential of a single charge which dominates the behavior at long distances. The second and third terms represent the dipole and quadrupole polarization of the core with *α*_d_ and *α*_q_, respectively. The parameters *A*_*l*_ and *ξ*_*l*_ depend on the orbital angular momentum and have to be selected according to the desired states. The concrete values for all the parameters are listed in ref. 65.

The wave function of the electron is computed under the assumption of spherical symmetry. In this case, the angular dependent part is solved by spherical harmonics, and only the radial part remains to be solved numerically. In order to achieve a balanced description for a large range of quantum numbers, and for the sake of higher accuracy, the radial Schrödinger equation was solved on a semi-infinite grid *via* an expansion in Chebyshev polynomials.^69^

### 2.2 The influence of the helium nanodroplet

In the next step we introduce repulsive effects due to the presence of helium in which the alkali metal ion is assumed to be immersed. An additional potential term was derived from the actual helium density distribution obtained from helium density functional theory based on a slightly modified version of the Orsay–Trento density functional.^70^,^71^ The free energy, a function of the helium density (*F*[*ρ*]), is minimized for an external potential describing the interaction with the alkali metal ion. The latter is approximated by a summation over pair potentials between the ion and a single He atom.

**Fig. 1:**
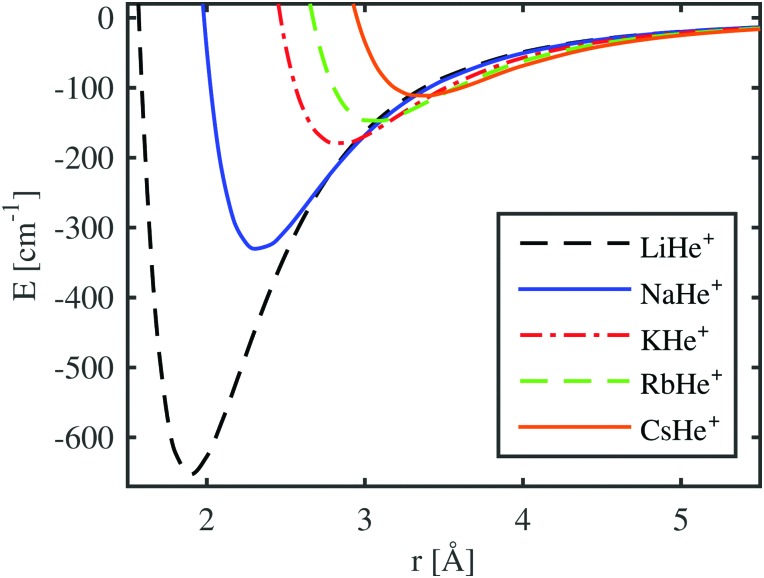
The CCSD(T) potential curves for several diatomic cations consisting of one alkali atom and one helium atom.

The necessary potential curves for all the alkali metal ions are calculated by coupled cluster theory with single, double and perturbative triple excitations as implemented in the Molpro software package.^72^,^73^ The He atom was described using the aug-cc-pV5Z basis set,^74^ and Li and Na atoms were described using the aug-cc-pCV5Z basis set.^75^ The effective core potentials of Lim *et al.*^76^ and the corresponding basis sets^76^,^77^ were used for K, Rb, and Cs. After a counterpoise correction,^78^ we obtain the potentials as shown in Fig. 1. The corresponding potential parameters are listed in Table 1 and are compared to previous studies. For Na and K, the values of Bellert *et al.*^64^ are listed, where the results of different experimental and theoretical approaches have been compared. The values for LiHe^+^ were taken from ref. 63, in which the ground state and several excited states have been calculated. In the case of Rb and Cs, we compare to a more recent work,^37^ which also included the treatment of superfluid helium droplets. The optimized densities determined with these potentials and their energies are presented in Section 3.

**Table 1:** A comparison of potential parameters of the CCSD(T) Ak^+^–He curves to previous studies. Our results were obtained for the isotopes ^4^He, ^7^Li, ^23^Na ^39^K, ^85^Rb, and ^133^Cs

Alkali metal	Li^*a*^	Li^63^	Na^*a*^	Na^64^	K^*a*^	K^64^	Rb^*a*^	Rb^37^	Cs^*a*^	Cs^37^
*R*_e_ [Å]	1.894	1.894	2.32	2.33	2.84	2.85	3.06	3.05	3.36	3.35
*D*_e_ [cm^–1^]	653	653	331	329	180	177	148	153	112	118
*ω*_e_ [cm^–1^]	276	276	160	157	104	100	89		72	
*ω*_e_ *x*_e_ [cm^–1^]	29	33	19		15		13		12	

^*a*^This work.

### 2.3 Coupling the helium density to the valence electron potential

With the helium density distributions evaluated in the previous step, we then compute the contribution to the electron potential. The approach of Cheng *et al.*^17^ is applied which is based on the work of Springett *et al.*,^79^ who tried to determine whether an excess electron can move freely in a non-polar fluid or is captured in a bubble. The energy of the excess electron consists of two parts: its kinetic energy and the potential energy due to polarization of the liquid. The Wigner–Seitz model is applied to compute these contributions. In this approach, the atoms are replaced by equivalent atomic spheres with a radius given by2
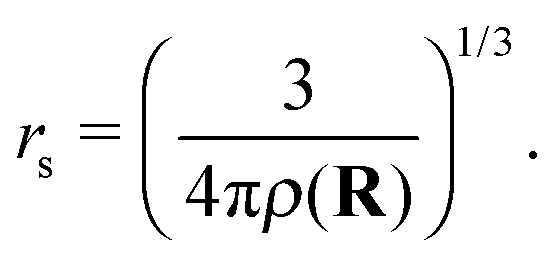
The wave vector of the electronic ground state can then be obtained with the boundary conditions for the Wigner–Seitz sphere, which leads to the transcendental equation:

3 tan[*k*_s_(*r*_s_ – *a*_c_)] = *k*_s_*r*_s_,

with a hard core scattering length *a*_c_ of 0.62 Å. The resulting kinetic energy term4
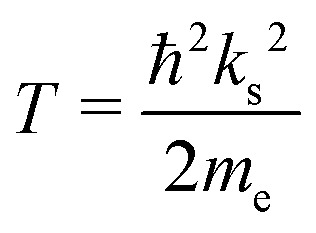
contains exchange repulsion *via* the orthogonalization of the electronic wave functions (boundary conditions). Following the work done in ref. 17, the second term that needs to be considered is the polarization potential, which is treated differently inside and outside the Wigner–Seitz sphere. Integrating over the region outside the sphere for a constant density results in the expression5
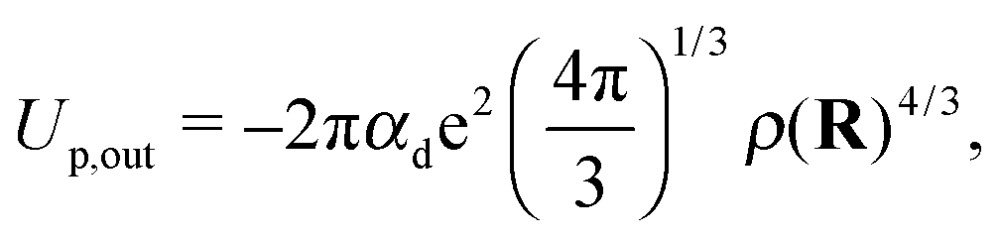
where a dipole polarizability of *α*_d_ = 0.204 Å^3^ was used in this work. In order to obtain the correct asymptotic behavior, the polarization potential inside the Wigner–Seitz sphere is given by6
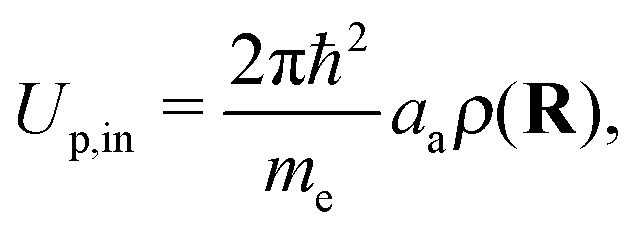
with *a*_a_ denoting the relevant scattering length due to the polarization potential. However, a value of 0 Å has shown the best agreement with experimental data,^18^ which renders this last contribution to the overall potential zero. In total, we obtain the following expression for the potential energy of a single electron embedded in a liquid helium environment *ρ*(**R**):7
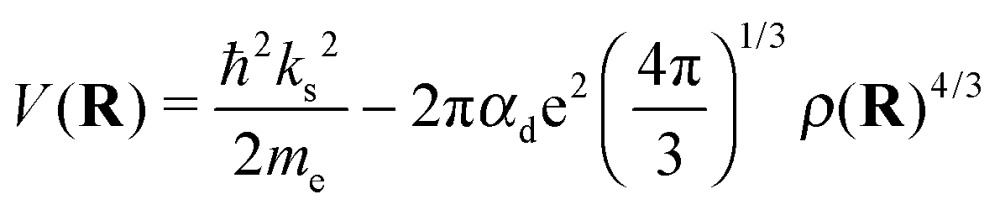



Note that eqn (7) is only valid if the helium density is weakly varying in space. This is not true for the given situation of an immersed ion where strong oscillations of the radial He density distribution are to be expected. The change in density results in different attractive contributions, because more or less atoms can be polarized at a specific distance. Mathematically, this can be included by an integration of the density differences for the cluster, resulting in the non-local potential8
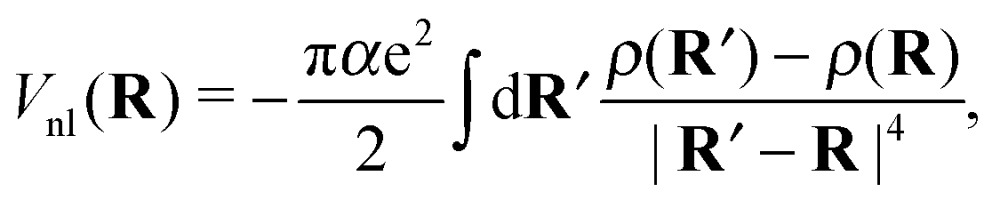
which – by construction – becomes zero if the density remains constant. The potential is increased by this expression (more repulsive) if the densities close by are lower. An attractive contribution is obtained if the density in the surrounding area is higher.

Additionally, we also consider the polarization of the helium by the ion. This is achieved by modifying the pseudopotential of the ionic core with the dielectric constant of the helium. The dielectric constant is computed from the He density using the Clausius–Mossotti relation^80^ with a polarizability of *α*_d_ = 0.204 Å^3^. A redistribution of charges in the helium due to the ion was neglected because it should be symmetric for a centered dopant.

## 3 Results and discussion

### 3.1 Accuracy of the Chebyshev method

**Fig. 2:**
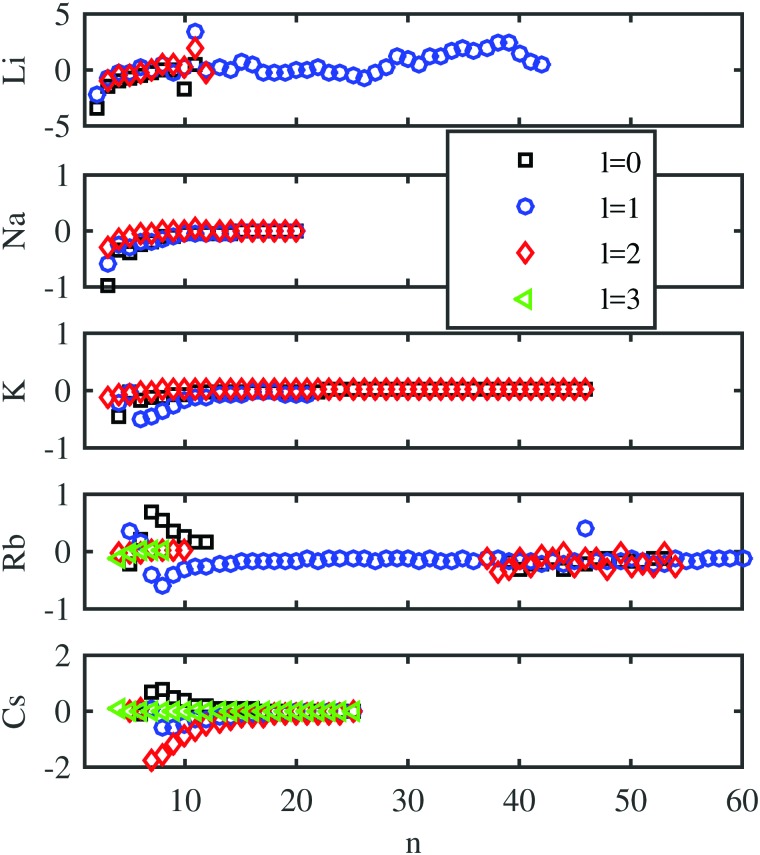
Differences between the binding energies in cm^–1^ determined by the Chebyshev method and those listed in the NIST database,^81^ calculated for all the alkali metal atoms as a function of the principal quantum number. Different marker shapes and colors indicate different angular momenta.

First, we evaluate the accuracy of the Chebyshev method for the unperturbed Rydberg states and compare it with the experimental results for bare atoms. The computed values are compared with the experimental energy levels taken from the NIST database^81^ in Fig. 2 for low and intermediate principal quantum numbers *n*. Deviations between the experimental and computed energies are below 1 cm^–1^ for Na, K, and Rb. They are slightly larger in the case of Li and Cs. In ref. 82 a similar comparison between the calculated and measured energy levels was presented, where no *l*-dependence of the error was observed. This lies in the nature of the ansatz of Callegari and Ancilotto,^82^ where the accuracy is dependent on the choice of appropriate basis functions. In our case, the accuracy can be steadily improved by increasing the number of Chebyshev polynomials.

Important parameters for the accuracy of the method are the mapping parameter *L*, the number of polynomials *M*, and the number of collocation points *N*. The mapping parameter *L* determines the distribution of the grid points. Strongly bound states with low quantum numbers are better described by small values of *L*, whereas large values of *L* are more suitable for high Rydberg states. A value of 800 a.u. has been chosen for our studies. The impact of *M* is obvious; with increasing number of Chebyshev polynomials (*M*) the error becomes smaller. Here, the correct treatment of the highest Rydberg states determines the number of polynomials necessary. The third parameter, the number of collocation points *N*, is chosen to be identical to the number of Chebyshev polynomials, which leads to a quadratic eigenvalue matrix that can be solved by standard eigenvalue solvers.

Another experimental reference, the Rydberg–Ritz formula of ref. 83, was used to determine the number of polynomials *M* required for accurate results. This analytical formula, with fit parameters derived from the experiment, allows the computation of Rydberg states with an accuracy of 10^–3^ cm^–1^ if the principal quantum number is larger than a specific value. The Rydberg–Ritz approach was also applied in the analysis of our previous Rb–He_*N*_ and Cs–He_*N*_ experiments, although with a lower accuracy because of the larger linewidths in the case of alkali–helium complexes compared to the free atom spectra.^60^ In our current computations, only spin-averaged results are provided. Accordingly, the weighted averages of the different spin states of the Rydberg–Ritz reference were used. A graphical comparison of the Rydberg–Ritz energies and those obtained *via* our Chebyshev method can be found in the ESI.† Tests show that at least 300 polynomials are needed for a correct description with a low *n*, but the additional gain in accuracy is minimal for larger sets. Non-vanishing deviations are observed for smaller principal quantum numbers where the strongly attractive part of the effective core potential plays a bigger role. The maximum deviation in this case is smaller than 4 cm^–1^ for all the alkali metal atoms except Rb. However, for rubidium, the Rydberg–Ritz formula is no longer a valid reference for small quantum numbers due to the inaccurate fitting parameters in this region. In contrast, the Rydberg–Ritz formula is a reliable reference for high principal quantum numbers which can be used to determine the number of required polynomials for states with a high *n*. For 800 Chebyshev polynomials, the deviations from the Rydberg–Ritz reference are below 0.1 cm^–1^ for states with principal quantum numbers between 15 and 80. There are additional figures in the ESI,† showing these trends. In the succeeding computations, 800 Chebyshev polynomials were applied.

### 3.2 Solvation energies for Ak^+^ ions in helium droplets

In this section we determine the energy gain of the total system by the immersion of the remaining alkali metal ion. This is achieved by the direct calculation of the helium density distribution and the corresponding total energy of the system *via* helium density functional theory (He-DFT) as a function of the distance between the alkali ion and the center of mass of the helium droplet. The solvation energy of an alkali metal ion, *E*_SOL_(Ak^+^), is defined as the energy difference between the fully immersed situation and the situation where the ion is located at an infinite distance from the droplet,

9 *E*_SOL_(Ak^+^) = *E*(He_*N*_ + Ak^+^) – *E*(He_*N*_).

**Fig. 3:**
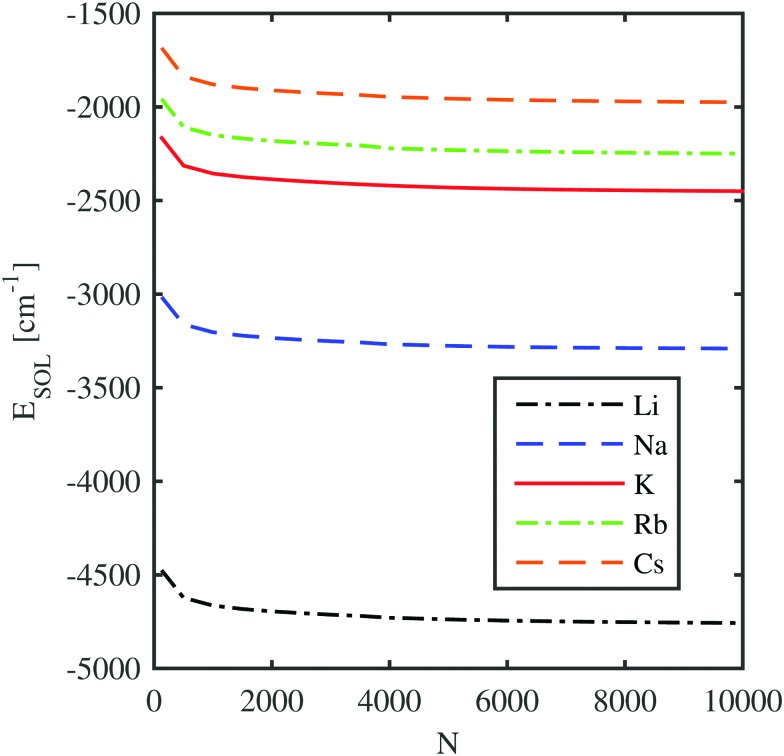
Solvation energies of different alkali metal ions submerged in He-clusters consisting of *N* helium atoms.

Using the *ab initio* potentials for the He–alkali metal ion interactions calculated in the previous section, we can generate external potentials for the He density *via* a pair potential summation and determine the total energy of the system for both geometries by a minimization procedure. This is done for all alkali metal ions and a selection of helium nanodroplets with various sizes, consisting of up to 10 000 He atoms. The dependence of the solvation energy on the droplet size is displayed in Fig. 3. Only for droplet sizes below 1000 helium atoms, the solvation energies differ significantly from the asymptotic bulk value. This finding already indicates a strong but local impact of the ion on the He density in the direct neighborhood.

### 3.3 He density distributions after immersion

**Fig. 4:**
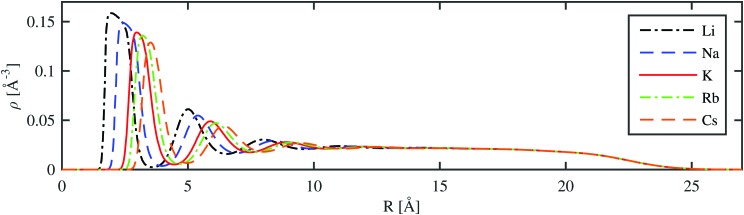
Radial helium density distribution of droplets consisting of 1000 He atoms with different alkali metal ions at the center (*R* = 0).

A more detailed picture is obtained by radial cuts through the helium density distribution for the situation where the alkali metal ion is fully submerged in the droplet. As shown in Fig. 4 for the case of a He_1000_ droplet, a series of radial density maxima or ‘shells’ is formed. By the integration of these density peaks, we obtain an estimate for the number of He atoms in each shell. Details of this analysis are provided in Table 2. We note that these numbers deviate slightly from previous studies due to obvious dependence on the chosen He–Ak^+^ potentials used to create the total potential for the He_*N*_–Ak^+^ interaction. The number of atoms in the first solvation shell is about 0.4 atoms lower than that reported in ref. 37 (19.2 and 21.4 atoms for Rb^+^ and Cs^+^, respectively). A different study by Galli *et al.*,^36^ where a path integral Monte Carlo technique was applied to small helium droplets, yielded energies per particle of 34.03, 23.53, and 22.90 K for He_128_ and Na^+^, K^+^, and Cs^+^, respectively. These values are close to our values listed in Table 2.

**Table 2:** Details of the calculated He densities, with *N*_*i*_ and *R*_*i*_ denoting the number of atoms and the radii of the *i*th solvation shell, respectively. The energy per helium atom [*E*/*N*] is given for three different cluster sizes. The energy gained by putting the alkali ion in a helium cluster is listed for 3000 He atoms. The maxima of the density considering all computed cluster sizes are given for the different alkali metals

	*N*_1_	*N*_2_	*R*_1_ [Å]	*R*_2_ [Å]	*E*/*N* [K] (128)	*E*/*N* [K] (3000)	*E*/*N* [K] (10 000)	*E*_SOL_ [K] (3000)	*ρ*_max_ [Å^–3^]
Li	11.9	30.9	3.6	6.6	–53.82	–7.66	–6.73	–6774	0.160
Na	14.1	34.4	4.0	7.0	–37.27	–6.95	–6.52	–4644	0.150
K	17.2	39.0	4.5	7.5	–27.88	–6.55	–6.40	–3460	0.140
Rb	18.8	41.2	4.7	7.7	–25.55	–6.45	–6.37	–3164	0.136
Cs	20.9	45.7	5.0	8.1	–22.47	–6.32	–6.33	–2775	0.130

The theoretical predictions of the shells will now be compared to stable structures in experiments. After photoionization of alkali metal atoms on superfluid helium droplets bare ions are emitted, but also ionic complexes with one alkali metal atom and several helium atoms. The photoion yield can be detected for different numbers of attached helium atoms and a drop in this signal indicates that larger structures are less stable. In ref. 11 and 40 a drop of the photoion yield was observed for Cs at about 15 to 17 attached helium atoms, about 5 atoms less than that in the first solvation shell given in Table 2. For the Rb ion, the drop was observed after 13 He atoms by Müller *et al.*^40^ and after 14 atoms by Theisen,^84^*i.e.* four or five atoms below our value. A drop of the ion yield after 9 and 12 atoms was observed in ref. 41 for Na and K, respectively. Again our theoretical estimates of the first solvation shell contain 5 additional atoms (see Table 2).

### 3.4 He-modified Rydberg states

In the final step, we compare the differences between the He-modified excited state energies and the Rydberg states of the free atom with the solvation energy of the ion. In a static approximate picture, the sign of this energy difference indicates the preferred position of the ion for a given Rydberg state: it can either reside inside or outside of the droplet. In order to do this, we combine the approaches of the previous sections to describe the excited states of the valence electron for a He-immersed alkali ion.

**Fig. 5:**
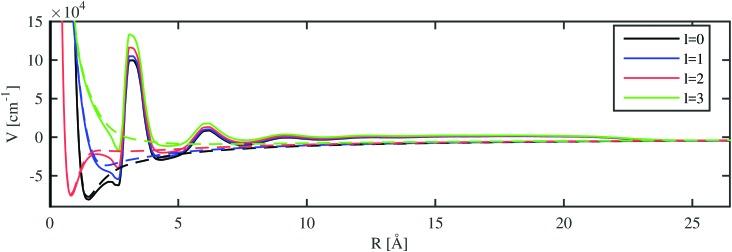
The *l*-dependent pseudopotentials (dashed lines) and resulting total potentials (continuous lines, including He density repulsion and polarization effects) are depicted for a fully submerged Rb^+^ ion inside a He_1000_ nanodroplet.

With the electron–helium interaction potential suggested in ref. 17, we can derive an additional potential term for the valence electron of the alkali metal atom from the helium densities calculated with the He-DFT approach. Eigenstates and eigenenergies are again obtained *via* numerical integration as described in Section 3.1. The model potential felt by the valence electron in the field of a Rb ion immersed in a He_1000_ nanodroplet is shown in Fig. 5. The modified potentials for the other alkali metal atoms are provided in the ESI.†

The inclusion of the extra potential term caused by the He density lifts the potential by about 1 eV for regions with a constant liquid density, *i.e.* between the inner density oscillations near the ion and the edge of the helium droplet. The parameters of the potential were adjusted by Cheng *et al.*^17^ to yield this experimentally determined value. For smaller distances, where the helium distribution shows a layered structure with peaks of high density, an oscillating perturbation is modulating the 1/*R* potential. Inside the local cavity formed around the alkali ion, the *l*-dependent repulsive parts of the atomic pseudopotentials are dominating. Note the unexpectedly steep left shoulder of the peak at about 3 Å, which is caused by the non-local contribution to the He–electron potential (see Section 2.3). In general, this correction leads to a lowering of the potential close to higher He densities and to an increase of the potential next to lower densities. As a consequence, the modified potential drops even below the unperturbed pseudopotential at about 2.5 Å. The same mechanism allows for bound states of electrons on the surface of helium as described in ref. 17. In the case of undoped, neutral helium droplets, an electron is only bound for very large droplets. Binding energies of 0.04 eV have been reported for droplets consisting of 5 × 10^5^ helium atoms.^16^

**Fig. 6:**
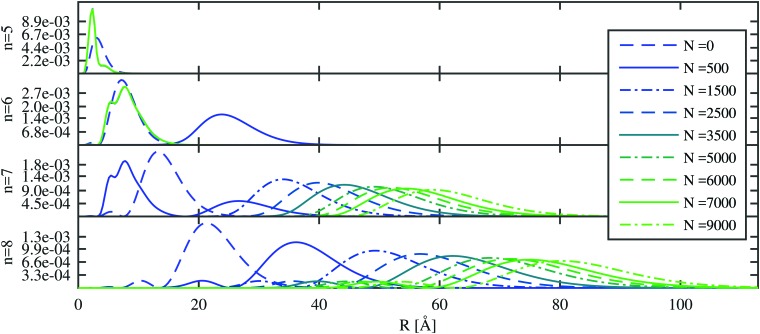
The probability density of the Rb valence electron is shown as a function of the radial distance for excited states with principal quantum numbers *n* = 5–8 and zero angular momentum. The unperturbed Rb density (*N* = 0) is compared to the results obtained after immersion into He_*N*_ of increasing size. The two states with the lowest principal quantum number (*n*) are located inside the droplet and their probability densities remain constant after a certain amount of helium has been added. States with higher principal quantum numbers are continuously pushed outward.

**Fig. 7:**
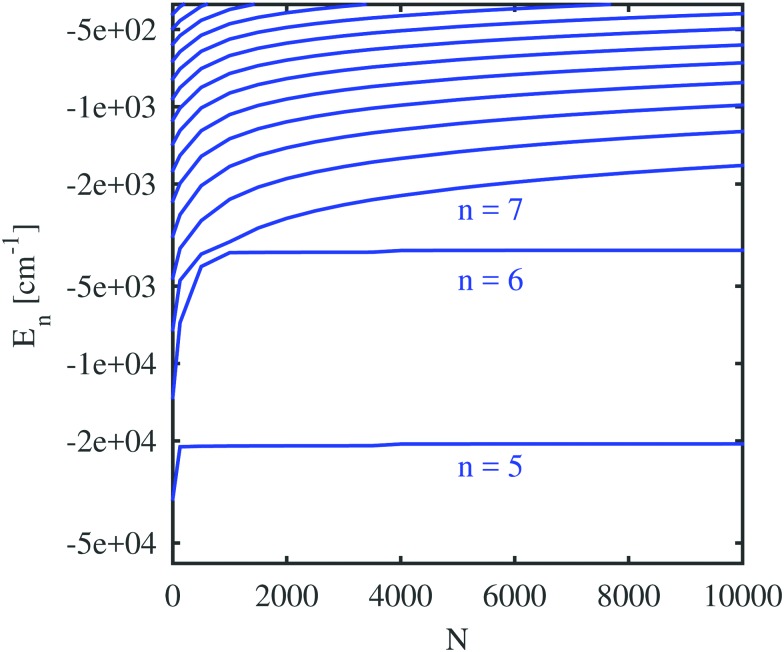
Rb valence electron energies for different principal quantum numbers (*n*), plotted as a function of the number of atoms forming the He cluster. *n* increases from bottom to top. The Rb ion is placed in the center of the droplet. Only states with zero orbital angular momentum are shown. Note that a logarithmic scale was used for the energy.

We start with the discussion of the radial probability densities for the valence electrons in the unperturbed and the He-affected states for helium droplets of increasing size as depicted in Fig. 6. The probability densities of almost all the states are continuously pushed outwards by the increasing amount of helium wrapping around the Ak^+^ ion. On average, the orbital radii show a strong increase at first and a linear dependence on the He_*N*_ radius for larger droplets. The latter is related to the number of helium atoms by *r*_He_ ∝ *N*^1/3^ (see ref. 60 and 85 for details). The binding energies of these states continuously decrease (see Fig. 7). Note that our theoretical model also permits states with the electron inside the helium droplet. Such states are obtained for orbital angular momenta of *l* = 0 and *l* = 1 and the lowest principal quantum numbers *n*. Their properties remained fairly constant after a certain amount of helium was added (*N* ≈ 1000). Accordingly, the expectation value of the radius for these states only changes for small numbers of helium atoms, as well as the binding energy (Fig. 7). However, we expect an immediate recombination of the electron and the ionic core followed by the expulsion of the neutral atom for these states.

Important for the interpretation of the ion behavior is the analysis of electron energies. Fig. 7 compares the unperturbed binding energies (*N* = 0) with those obtained when submerged in helium droplets of various sizes. Several states of the Rb valence electron are depicted with an orbital angular momentum of *l* = 0 (higher angular momenta show a very similar dependence and are included in the ESI†) and different principal quantum numbers. The electronic states with low principal quantum numbers show an immediate decrease in the binding energy as soon as He is added, but become quickly independent of the actual droplet size. Again, this indicates that the corresponding radial eigenfunctions have their maxima within the range of the density fluctuations that occur around the alkali ion and are therefore barely affected by the actual amount of helium at larger radial distances. States with higher principal quantum numbers, on the other hand, show a steady decrease in the binding energy with droplet size since they are indeed located outside of the droplet. This behavior is similar to the one predicted by Golov and Sekatskii for an electron bound to an ionized helium cluster.^12^,^13^ A further comparison of the various curves reveals that the states of higher principal quantum numbers show a less pronounced blueshift with droplet size, which is an obvious consequence of their larger orbits and smaller overlap with helium.

**Fig. 8:**
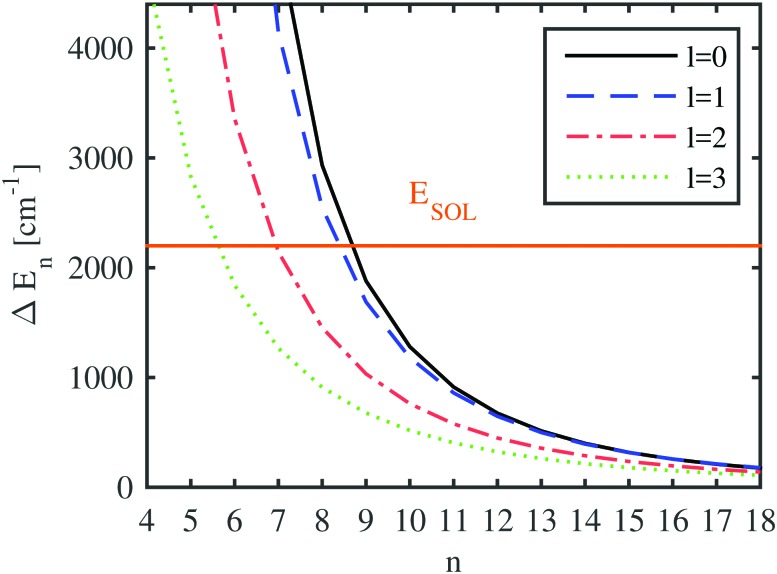
The energy difference between the states of the electron bound to the free Rb ion and to an ion submerged in a helium droplet consisting of 3000 atoms (Δ*E*) is plotted for different principal quantum numbers and orbital angular momenta. The negative solvation energy is also included (–*E*_SOL_). Therefore, in the case of energy differences larger than the solvation energy, a position outside the droplet is favored, otherwise an alkali metal ion surrounded by helium is preferred.

With the computed He-modified eigenenergies, we can finally attempt to give estimations for the behavior of the Ak^+^ ion by comparing the blueshift of a certain electronically excited state with quantum numbers *n*, *l* (*i.e.* the additional energy cost for pushing the electron in this state further outside) to the energy gained by the solvation of the alkali ion. This is done in Fig. 8, where the energy differences between the unperturbed and the perturbed electronic states of Rb are plotted as a function of the principal quantum number, together with the corresponding solvation energy for a Rb^+^ ion. A helium droplet consisting of 3000 atoms has been selected for depiction. The differences in the electronic energies approach zero for high Rydberg states due to the vanishing overlap of the wave function with area occupied by helium. Depending on the angular momentum of the electronic state, the curve denoting the difference in electronic energies (*i.e.* the blueshift) intersects the line indicating the solvation energy for principal quantum numbers between 6 and 9. This is where our static model suggests a qualitative rearrangement of the system geometry: for energy differences larger than the solvation energy, a position of the alkali atom on the surface of the droplet is favored. However, as soon as this blueshift of the excited states is smaller than the solvation energy, the geometry becomes preferred where the ion is fully immersed while the valence electron keeps orbiting the He nanodroplet.

Obviously, the location of the crossover regime is dependent on the droplet size due to the size-dependent solvation energy and the impact of the different He density distributions on the potential felt by the valence electron. This influence is graphically documented in the ESI.† However, the minimal shift to higher principal quantum numbers with increasing cluster size is almost negligible over the experimentally relevant droplet sizes due to the comparably large orbital radii of these excited states in comparison to the droplet radius.

### 3.5 Comparison to experiments

Alkali metal atoms in their electronic ground state are known to reside on the surface of the helium droplet.^9^ Upon excitation, they can remain on the droplet, detach from it or go inside the droplet, depending on the He-interaction potential in the corresponding electronically excited state. We note that this configuration of having a neutral alkali metal atom attached to the surface of a helium droplet is the starting point for most spectroscopic investigations in helium nanodroplet isolation spectroscopy. Therefore, from the experimental perspective, our computational approach is making the strong and yet unconfirmed assumption of an immersion triggered by electronic excitation. We are aware that an in-depth analysis of such a process necessitates a fully dynamic treatment, but our static approach can provide reasonable estimates for transition quantum numbers. In this sense, our study takes an alternative point of view when compared to previous treatments within the so-called ‘diatomic’ picture, where the interaction of a single atom with He_*N*_ is treated like a diatomic molecule.

Interaction potentials of the latter type, describing the first few excited states of a surface-bound alkali atom, have already been calculated for the whole Ak series.^45^,^82^,^86^ These potential curves also comprise information about line shifts for the low-lying states. In most cases they are blue shifted, which indicates a more repulsive potential in the excited state, leading to detachment from the dopant upon exitation. Exceptions are the 5^2^P_1/2_ state of Rb and the 6^2^P_1/2_ state of Cs, where the ‘diatomic’ interaction potentials in the excited states are very similar to the ground state potential, leading to smaller line shifts and the ability to excite the alkali atom without causing detachment.^10^,^11^

However, in contrast to the blueshift of the lower electronically excited states, the ionization threshold of alkali atoms is lowered when they are bound to the surface of superfluid helium nanodroplets.^87^ This experimental finding coincides with our observation of a transition from blue-shifted lower excited states and a preference for ejection upon excitation to red-shifted higher excited states with a preference for ion submersion. Experiments of our group were dedicated to the concrete localization of such a transition from blue- to red-shifted excitations with increasing principal quantum number by a measurement of Rydberg series for alkali-metal-doped He nanodroplets.^60^,^61^,^85^

Table 3 consists of the results of our current computational approach and compares the proposed quantum number regimes for ion immersion to results from experiments. We list the lowest principal quantum number (for s, p, d and f angular momentum) with a negative energy balance, *i.e.* a situation where energy gain by immersion starts to overcompensate the blueshift of the corresponding state. Experimental values, on the other hand, are the lowest principal quantum numbers of the measured Rydberg states for which a redshift is observed. As already mentioned, the ground-state alkali metal atom is located on the surface in the experimental studies. Therefore, a diatomic picture with an interatomic axis from the alkali metal atom to the center of the droplet is appropriate. In this picture, the electronic states can be labeled with respect to the angular momentum projection onto the axis, which is also included in the table. In general, the experimental principal quantum numbers for immersion seem to be slightly larger, in particular for lower values of the orbital angular momentum projection. To some extent this deviation may be related to the diatomic nature of the system before excitation and the shape and orientation of the orbitals with different symmetry. In particular, low Λ states (*e.g.* Σ) will have more overlap with the He_*N*_ giving rise to an increased repulsive interaction, which manifests in an increased *n* for the transition from blue to redshift. Furthermore, our results reflect the trend found in the experiments that with increasing size of the alkali dopant, the transition is shifted to a high *n*. There is also agreement with the experiment that with increasing *l* the transition quantum number shifts to a lower *n*.^61^

**Table 3:** A comparison of ‘transition’ quantum numbers where the electronically excited alkali atom is supposed to sink into the droplet, predicted from theory, to experimental estimates based on a change from blueshift to redshift in the excitation spectra for the originally surface-bound alkali metal atoms. Note that the system is not spherically symmetric if the alkali atom is residing on the surface. In this case, also the projection of the angular momentum becomes important and is therefore given in brackets (Σ, Π, Δ). References are given in the last column

	Theory				*N*	Experiment				*N*	Ref.
	s	p	d	f		s	p	d	f		
Li	5	5	5		6000	7 (Σ)	5 (Π)			6000	56
Na	7	6	6		6000	>6	>5	>5		6100	57
K	8	8	6		5000						
Rb	9	9	8	6	5000	14 (Σ)	13 (Σ), 10 (Π)	11 (Σ/Π), 8 (Δ)	6	5000	59, 60 and 85
Cs	11	10	9	7	7000	13 (Σ)	12 (Σ), 10 (Π)	13 (Π), 10 (Δ)		7500	61

Leal *et al.*^37^ discussed the possibility that an alkali ion is not absorbed after ionization on the surface of the droplet, but a solvation shell is formed around the ion instead and both detach from the helium droplet. However, this behavior was only found for a small helium droplet containing about 1000 atoms and was disproven for large droplets.^11^,^27^ Furthermore, the time needed for the formation of a solvation shell is only 10–20 ps. This also makes the submersion process plausible at least for the larger droplets studied in this work, especially when it is compared to the microsecond lifetimes for the Rydberg states of alkali metal atoms on the nanodroplets.^58^

The stability of the system after submersion of the ion core cannot be estimated within the chosen static picture, but it is our assumption that lifetimes will be dominated by indirect recombination processes of the electron through interactions with the helium surface. The picture of an almost freely moving dopant inside the helium droplet, as it has been suggested for neutral metal atoms,^88^ is not applicable here due to the very strong interaction of the ionic core with the helium environment which even leads to freezing of He near the ion.

## 4 Conclusions

We investigated the electronic excitations of alkali metal atoms (Li, Na, K, Rb, Cs) immersed in helium nanodroplets with sizes ranging from 128 to 10 000 He atoms. Helium density functional theory has been applied in order to obtain realistic He density distributions after the submersion of an alkali metal ion. The pressure on the helium droplet by the valence electron^8^ was neglected here, because it only slightly modifies the density distribution and does not alter our results significantly, especially for spread-out Rydberg states and large helium droplets. Using the He-density distributions, we could derive an additional potential term which we added to a standard pseudopotential ansatz used for the description of valence-electron eigenstates. As a side product we also obtained solvation energies for all alkali ion dopants. Both ingredients, the modified potential felt by the valence electron in a highly excited state of the neutral atom and the solvation energies for the electron-stripped singly charged Ak^+^ ions, were then used to estimate ‘transition' principal quantum numbers for each alkali metal atom where submersion of the neutral alkali metal atom should occur upon electronic excitation. In our model, this turnover point is reached when the blueshift of the Rydberg state, caused by the He perturbation, is smaller than the gain in energy by solvation of the alkali metal ion. Our predictions indicate a stable system where the Ak^+^ ion sits inside the droplet while the valence electron remains orbiting outside of the droplet, for quantum numbers larger than 5 to 11, depending on the angular momentum and the specific alkali metal atom. Recent experiments yielded slightly higher principal quantum numbers for the calculated switch from blue to redshift, which might be caused by an additional barrier for the submersion which cannot be captured within the proposed static picture. However, a more detailed analysis based on time-dependent studies might be able to clarify this in future studies.

## Supplementary Material

Supplementary information

## References

[cit1] Platzman P. M., Dykman M. I. (1999). Science.

[cit2] Dykman M. I., Platzman P. M., Seddighrad P. (2003). Phys. Rev. B: Condens. Matter Mater. Phys..

[cit3] Lyon S. A. (2006). Phys. Rev. A: At., Mol., Opt. Phys..

[cit4] Schuster D. I., Fragner A., Dykman M. I., Lyon S. A., Schoelkopf R. J. (2010). Phys. Rev. Lett..

[cit5] Yang G., Fragner A., Koolstra G., Ocola L., Czaplewski D. A., Schoelkopf R. J., Schuster D. I. (2016). Phys. Rev. X.

[cit6] Collin E., Bailey W., Fozooni P., Frayne P. G., Glasson P., Harrabi K., Lea M. J., Papageorgiou G. (2002). Phys. Rev. Lett..

[cit7] Stienkemeier F., Lehmann K. K. (2006). J. Phys. B: At., Mol. Opt. Phys..

[cit8] Ancilotto F., Pi M., Mayol R., Barranco M., Lehmann K. K. (2007). J. Phys. Chem. A.

[cit9] Callegari C. and Ernst W. E., in Handbook of High-Resolution Spectroscopy, ed. M. Quack and F. Merkt, John Wiley & Sons, Chichester, 2011, pp. 1551–1594.

[cit10] Auböck G., Nagl J., Callegari C., Ernst W. E. (2008). Phys. Rev. Lett..

[cit11] Theisen M., Lackner F., Ernst W. E. (2011). J. Chem. Phys..

[cit12] Golov A., Sekatskii S. K. (1993). Z. Phys. D: At., Mol. Clusters.

[cit13] Golov A., Sekatskii S. K. (1994). Phys. B.

[cit14] Lerner P. B., Sokolov I. M. (1986). JETP Lett..

[cit15] Chalupa J. (1982). Solid State Commun..

[cit16] Krishna M. V. R., Whaley K. B. (1988). Phys. Rev. B: Condens. Matter Mater. Phys..

[cit17] Cheng E., Cole M. W., Cohen M. H. (1994). Phys. Rev. B: Condens. Matter Mater. Phys..

[cit18] Cheng E., Cole M. W., Cohen M. H. (1994). Phys. Rev. B: Condens. Matter Mater. Phys..

[cit19] Fowler W. B., Dexter D. L. (1968). Phys. Rev..

[cit20] Sekatskii S. K. (1997). JETP Lett..

[cit21] Grau V., Barranco M., Mayol R., Pi M. (2006). Phys. Rev. B: Condens. Matter Mater. Phys..

[cit22] Mateo D., Jin D. F., Barranco M., Pi M. (2011). J. Chem. Phys..

[cit23] Barragan J., Mateo D., Pi M., Salvat F., Barranco M., Maris H. J. (2013). J. Low Temp. Phys..

[cit24] Guo W., Jin D., Seidel G. M., Maris H. J. (2009). Phys. Rev. B: Condens. Matter Mater. Phys..

[cit25] Maris H. J. (2008). J. Phys. Soc. Jpn..

[cit26] Xie Z., Wei W., Yang Y., Maris H. J. (2014). J. Exp. Theor. Phys..

[cit27] Theisen M., Lackner F., Ernst W. E. (2010). Phys. Chem. Chem. Phys..

[cit28] Sebastianelli F., Bodo E., Baccarelli I., Di Paola C., Gianturco F. A., Yurtsever M. (2006). Comput. Mater. Sci..

[cit29] Marinetti F., Uranga-Pinia L. I., Coccia E., Lopez-Duran D., Bodo E., Gianturco F. A. (2007). J. Phys. Chem. A.

[cit30] Marinetti F., Coccia E., Bodo E., Gianturco F. A., Yurtsever E., Yurtsever M., Yildirim E. (2007). Theor. Chem. Acc..

[cit31] Galli D. E., Buzzacchi M., Reatto L. (2001). J. Chem. Phys..

[cit32] Buzzacchi M., Galli D. E., Reatto L. (2001). Phys. Rev. B: Condens. Matter Mater. Phys..

[cit33] Rossi M., Verona M., Galli D. E., Reatto L. (2004). Phys. Rev. B: Condens. Matter Mater. Phys..

[cit34] Slavicek P., Lewerenz M. (2010). Phys. Chem. Chem. Phys..

[cit35] Paolini S., Ancilotto F., Toigo F. (2007). J. Chem. Phys..

[cit36] Galli D. E., Ceperley D. M., Reatto L. (2011). J. Phys. Chem. A.

[cit37] Leal A., Mateo D., Hernando A., Pi M., Barranco M., Ponti A., Cargnoni F., Drabbels M. (2014). Phys. Rev. B: Condens. Matter Mater. Phys..

[cit38] Fiedler S. L., Mateo D., Aleksanyan T., Eloranta J. (2012). Phys. Rev. B: Condens. Matter Mater. Phys..

[cit39] Tiggesbaumker J., Stienkemeier F. (2007). Phys. Chem. Chem. Phys..

[cit40] Müller S., Mudrich M., Stienkemeier F. (2009). J. Chem. Phys..

[cit41] An der Lan L., Bartl P., Leidlmair C., Jochum R., Denifl S., Echt O., Scheier P. (2012). Chem. – Eur. J..

[cit42] Mudrich M., Stienkemeier F. (2014). Int. Rev. Phys. Chem..

[cit43] Bünermann O., Droppelmann G., Hernando A., Mayol R., Stienkemeier F. (2007). J. Phys. Chem. A.

[cit44] Nagl J., Auböck G., Hauser A. W., Allard O., Callegari C., Ernst W. E. (2008). Phys. Rev. Lett..

[cit45] Lackner F., Poms J., Krois G., Pototschnig J. V., Ernst W. E. (2013). J. Phys. Chem. A.

[cit46] Loginov E., Drabbels M. (2014). J. Phys. Chem. A.

[cit47] Pifrader A., Allard O., Auböck G., Callegari C., Ernst W. E., Huber R., Ancilotto F. (2010). J. Chem. Phys..

[cit48] Fechner L., Gruner B., Sieg A., Callegari C., Ancilotto F., Stienkemeier F., Mudrich M. (2012). Phys. Chem. Chem. Phys..

[cit49] Theisen M., Lackner F., Ancilotto F., Callegari C., Ernst W. E. (2011). Eur. Phys. J. D.

[cit50] Reho J., Callegari C., Higgins J., Ernst W. E., Lehmann K. K., Scoles G. (1997). Faraday Discuss..

[cit51] Reho J., Higgins J., Lehmann K. K., Scoles G. (2000). J. Chem. Phys..

[cit52] Reho J., Higgins J., Callegari C., Lehmann K. K., Scoles G. (2000). J. Chem. Phys..

[cit53] Brühl F. R., Trasca R. A., Ernst W. E. (2001). J. Chem. Phys..

[cit54] Droppelmann G., Bünermann O., Schulz C. P., Stienkemeier F. (2004). Phys. Rev. Lett..

[cit55] Hofer A., Moroshkin P., Nettels D., Ulzega S., Weis A. (2006). Phys. Rev. A: At., Mol., Opt. Phys..

[cit56] Lackner F., Krois G. and Ernst W. E., TBA, 2017, Lithium Atoms on Helium Nanodroplets: Rydberg Series and Ionization Dynamics, in preparation. 10.1063/1.5004543 29141430

[cit57] Loginov E., Callegari C., Ancilotto F., Drabbels M. (2011). J. Phys. Chem. A.

[cit58] Loginov E., Drabbels M. (2011). Phys. Rev. Lett..

[cit59] Lackner F., Krois G., Koch M., Ernst W. E. (2012). J. Phys. Chem. Lett..

[cit60] Lackner F., Krois G., Ernst W. E. (2013). Mol. Phys..

[cit61] Lackner F., Krois G., Theisen M., Koch M., Ernst W. E. (2011). Phys. Chem. Chem. Phys..

[cit62] Bünermann O., Kornilov O., Haxton D. J., Leone S. R., Neumark D. M., Gessner O. (2012). J. Chem. Phys..

[cit63] Soldan P., Kraemer W. P. (2012). Chem. Phys..

[cit64] Bellert D., Breckenridge W. H. (2002). Chem. Rev..

[cit65] Bardsley J. N. (1974). Case Stud. At. Phys..

[cit66] Fuentealba P., Preuss H., Stoll H., von Szentpaly L. (1982). Chem. Phys. Lett..

[cit67] von Szentpaly L., Fuentealba P., Preuss H., Stoll H. (1982). Chem. Phys. Lett..

[cit68] Marinescu M., Sadeghpour H. R., Dalgarno A. (1994). Phys. Rev. A: At., Mol., Opt. Phys..

[cit69] Boyd J. P., Rangan C., Bucksbaum P. H. (2003). J. Comput. Phys..

[cit70] Dalfovo F., Lastri A., Pricaupenko L., Stringari S., Treiner J. (1995). Phys. Rev. B: Condens. Matter Mater. Phys..

[cit71] Ancilotto F., Barranco M., Caupin F., Mayol R., Pi M. (2005). Phys. Rev. B: Condens. Matter Mater. Phys..

[cit72] Werner H.-J., Knowles P. J., Knizia G., Manby F. R., Schütz M., Celani P., Korona T., Lindh R., Mitrushenkov A., Rauhut G., Shamasundar K. R., Adler T. B., Amos R. D., Bernhardsson A., Berning A., Cooper D. L., Deegan M. J. O., Dobbyn A. J., Eckert F., Goll E., Hampel C., Hesselmann A., Hetzer G., Hrenar T., Jansen G., Köppl C., Liu Y., Lloyd A. W., Mata R. A., May A. J., McNicholas S. J., Meyer W., Mura M. E., Nicklass A., O'Neill D. P., Palmieri P., Peng D., Pflüger K., Pitzer R., Reiher M., Shiozaki T., Stoll H., Stone A. J., Tarroni R., Thorsteinsson T. and Wang M., MOLPRO, version 2012.1, a package of ab initio programs, 2012, see http://www.molpro.net.

[cit73] Hampel C., Peterson K. A., Werner H. J. (1992). Chem. Phys. Lett..

[cit74] Woon D. E., Dunning T. H. (1994). J. Chem. Phys..

[cit75] Prascher B. P., Woon D. E., Peterson K. A., Dunning T. H., Wilson A. K. (2011). Theor. Chem. Acc..

[cit76] Lim I. S., Schwerdtfeger P., Metz B., Stoll H. (2005). J. Chem. Phys..

[cit77] The basis sets were used in decontracted form and have been extended by functions with the following exponents: K[s: 0.0037, 0.0017; p:0.0016, 0.00062; d: 4.34, 0.011; f: 0.0296]; Rb[s: 0.0036, p: 0.0042, d: 0.0116, 2.860, f: 0.0624 g: 0.33]

[cit78] Boys S. F., Bernadi F. (1970). Mol. Phys..

[cit79] Springett B. E., Jortner J., Cohen M. H. (1968). J. Chem. Phys..

[cit80] Donnelly R. J., Barenghi C. F. (1998). J. Phys. Chem. Ref. Data.

[cit81] Ralchenko Y., Kramida A., Reader J. and NIST ASD Team, NIST Atomic Spectra Database (version 5.4), National Institute of Standards and Technology, Gaithersburg, MD, 2015.

[cit82] Callegari C., Ancilotto F. (2011). J. Phys. Chem. A.

[cit83] Lorenzen C. J., Niemax K. (1983). Phys. Scr..

[cit84] Theisen M., Doctoral thesis, Graz Universtiy of Technology, 2011.

[cit85] Lackner F., Doctoral thesis, Graz Universtiy of Technology, 2012.

[cit86] Nakayama A., Yamashita K. (2001). J. Chem. Phys..

[cit87] Theisen M., Lackner F., Krois G., Ernst W. E. (2011). J. Phys. Chem. Lett..

[cit88] Hauser A. W., Volk A., Thaler P., Ernst W. E. (2015). Phys. Chem. Chem. Phys..

