# Supplementary material for: Rydberg states of alkali atoms on superfluid helium nanodroplets: inside or outside?
Source: Phys Chem Chem Phys. 2017 May 16;19(22):14718–28. doi: 10.1039/c7cp02332d (PMC5708348; doi:10.1039/c7cp02332d)
Supplement: Supplementary file 1 [file CP-019-C7CP02332D-s001.pdf]

Rydberg states of alkali atoms on superfluid helium nanodroplets: Inside or outside? - Electronic Supplementary Information

Johann V. Pototschnig, Florian Lackner, Andreas W. Hauser,* and Wolfgang E. Ernst†

Institute of Experimental Physics, Graz University of Technology,

Petersgasse 16, A-8010 Graz, Austria.

(Dated: May 11, 2017)

Introduction

This supplementary information contains additional data concerning our article entitled: 'Rydberg states of alkali atoms on superfluid helium nanodroplets: Inside or outside?' published in PCCP. Section I addresses questions of accuracy regarding the Chebyshev ansatz for the electronically excited states and investigates how many states can be described by a specific number of polynomials. Section II illustrates the impact of the helium density effects on the unperturbed pseudopotentials of all alkali metal atoms investigated. The remaining sections of this manuscript provide a more complete analysis and comparison of all alkali metal atoms; in the main article only the Rb results are discussed for brevity. We start with the probability densities in section III. The dependence of state energies and the mean orbit radii on the droplet size are presented in section IV. In the last section (V) we compare the solvation energies for the alkali metal ions to differences in the binding energies obtained for the excited states of the free alkali metal atoms and those immersed into a helium droplet.

* andreas.w.hauser@gmail.com

† wolfgang.ernst@tugraz.at

CONTENTS

I. Deviations 3

II. Potentials 6

III. Probability densities 9

IV. Energies and mean radii for the valence electron in the combined potential 12

V. Intersection between solvation energy and binding energy 17

References 20

I. DEVIATIONS

In this section we take a closer look at the accuracy of the spectral method. The reference for the results is the Rydberg-Ritz formula of Ref. 1, a fit to experimental results. Only the results for an orbital angular momentum of zero are shown in the figures 1 to 5; states with higher angular momentum show a similar behavior. Details of the method can be found in the main text.

An increase of the number of Chebyshev polynomials (M) leads to a larger number of states (all markers). The number of accurate states (i.e. states with errors below 0.1 cm^{-1}) grows with M. However, for several low lying states the agreement between calculated and fitted values does not improve with M. In part, this stems from the errors in the Rydberg-Ritz parameters, especially for Rb. In general, for low lying states the NIST-values[2] seem more reliable. Therefore, a comparison with NIST data is given in the main manuscript, showing that the deviations are bellow 5 cm^{-1} even for strongly bound states (binding energies of about 30000 cm^{-1}).

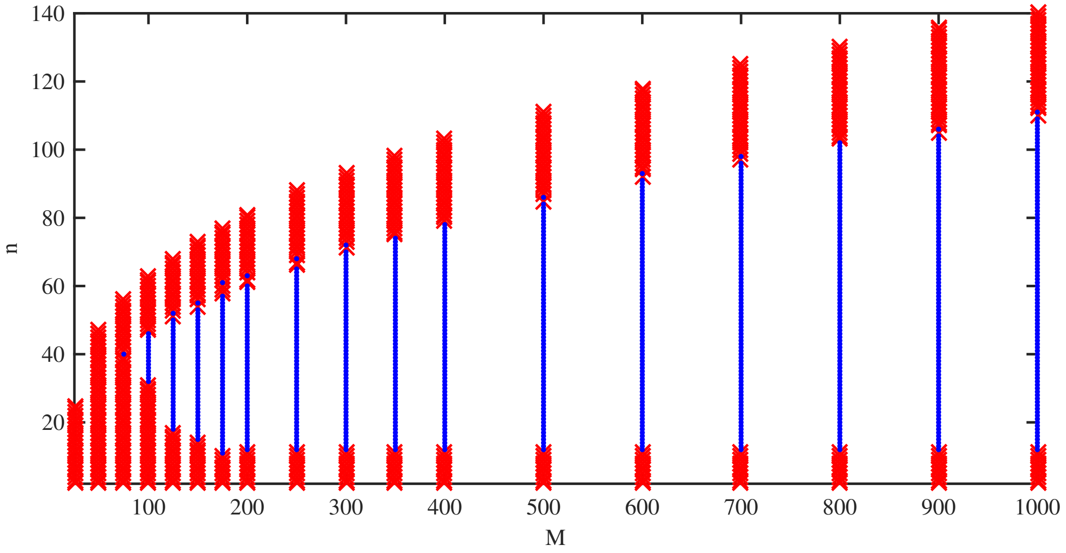

FIG. 1. The number of determined bound states and their accuracy for different numbers of Chebyshev polynomials (M) are depicted for s-states of Li. A red x indicates that a bound state was found for a specific principal quantum number (n). Blue dots are used if the energy deviates by less then 0.1 cm^{-1} from the Rydberg-Ritz values.[1]

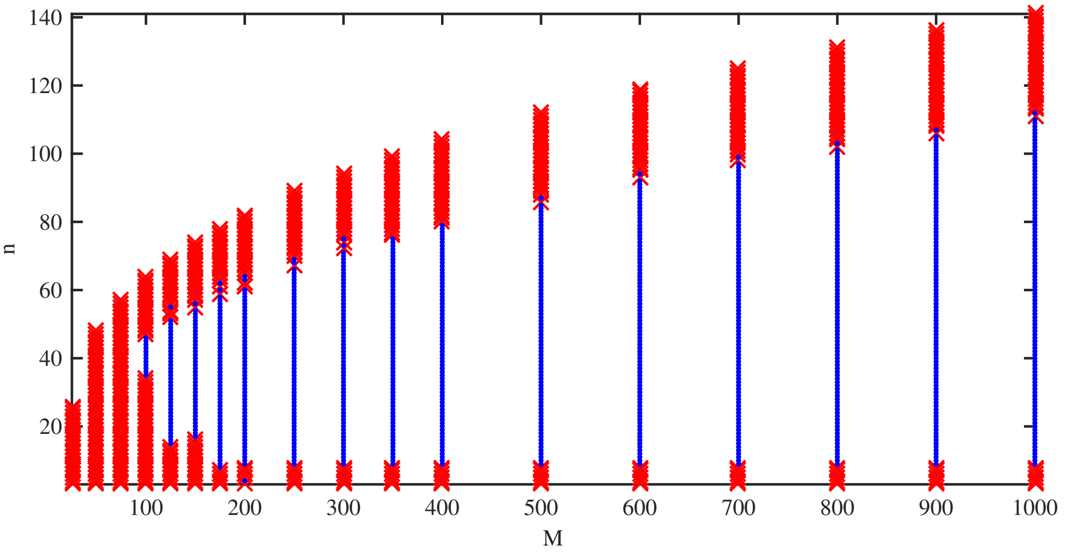

FIG. 2. The number of determined bound states and their accuracy for different numbers of Chebyshev polynomials (M) are depicted for s-states of Na. A red x indicates that a bound state was found for a specific principal quantum number (n). Blue dots are used if the energy deviates less then 0.1 cm^{-1} from the Rydberg-Ritz values.[1]

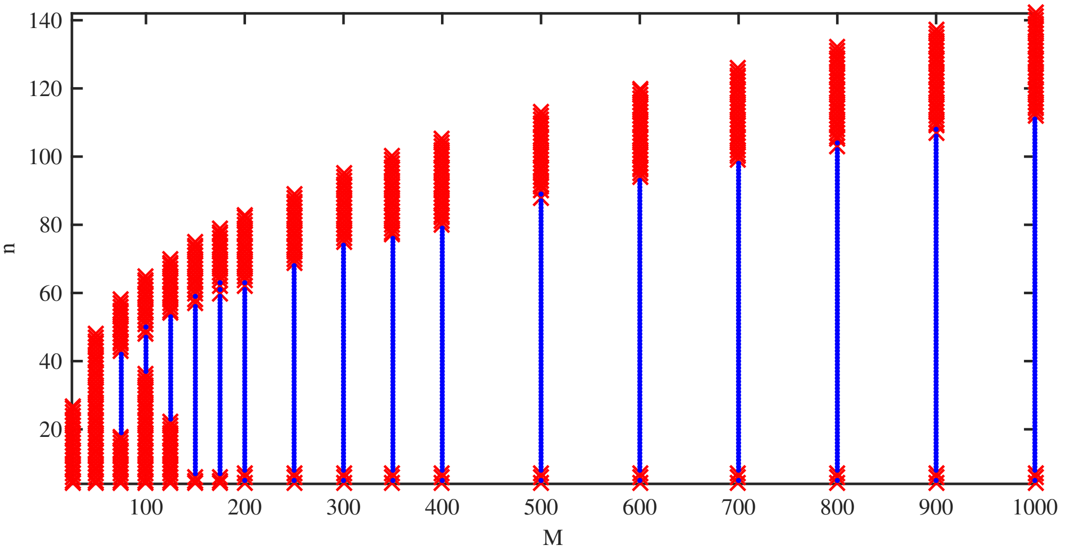

FIG. 3. The number of determined bound states and their accuracy for different numbers of Chebyshev polynomials (M) are depicted for s-states of K. A red x indicates that a bound state was found for a specific principal quantum number (n). Blue dots are used if the energy deviates less then 0.1 cm^{-1} from the Rydberg-Ritz values.[1]

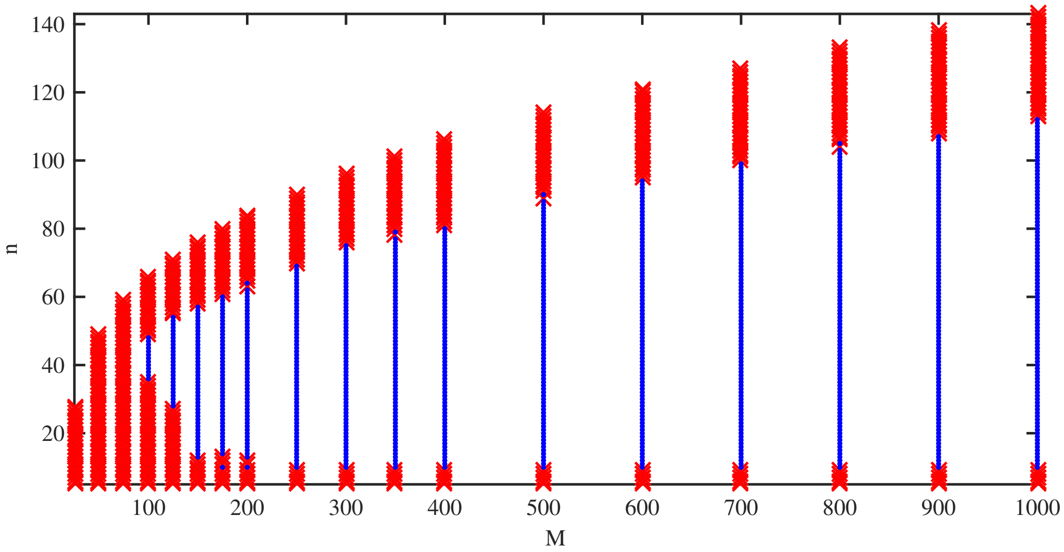

FIG. 4. The number of determined bound states and their accuracy for different numbers of Chebyshev polynomials (M) are depicted for s-states of Rb. A red x indicates that a bound state was found for a specific principal quantum number (n). Blue dots are used if the energy deviates less then 0.1 cm^{-1} from the Rydberg-Ritz values.[1]

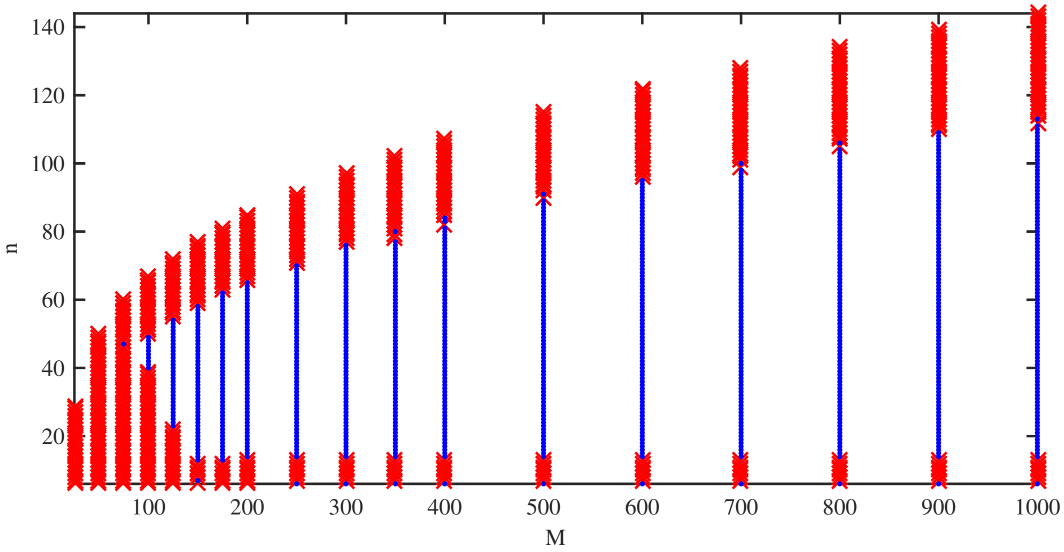

FIG. 5. The number of determined bound states and their accuracy for different numbers of Chebyshev polynomials (M) are depicted for s-states of Cs. A red x indicates that a bound state was found for a specific principal quantum number (n). Blue dots are used if the energy deviates less then 0.1 cm^{-1} from the Rydberg-Ritz values.[1]

II. POTENTIALS

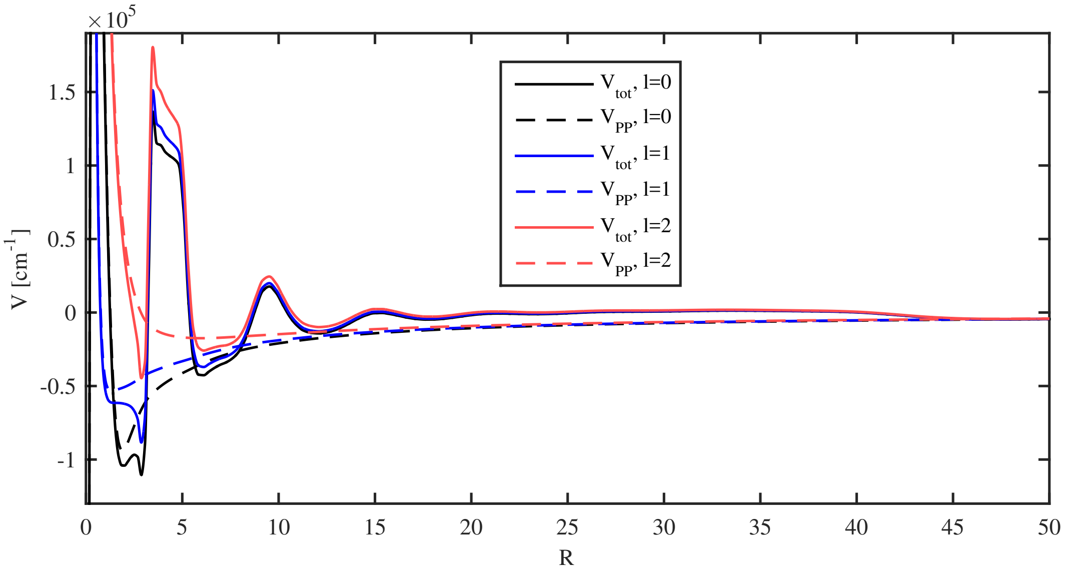

FIG. 6. The l-dependent total potential for the valence electron of a Li atom immersed into a helium droplet consisting of 1000 He atoms (solid line). The corresponding unperturbed pseudopotentials for Li are shown as dashed lines for comparison.

The impact of the helium density on the potential felt by the valence electron is documented in Figures 6, 7, 8, 9, and 10. The perturbation of the pseudopotentials was determined from the He densities obtained for the immersed alkali based on the formula of Cheng *et al.*[3, 4] (see main text). The non-local contribution tends to further reduce the potential depth in areas of higher He density, which can be interpreted as additional binding of an electron to the local helium surface by mirror charges.

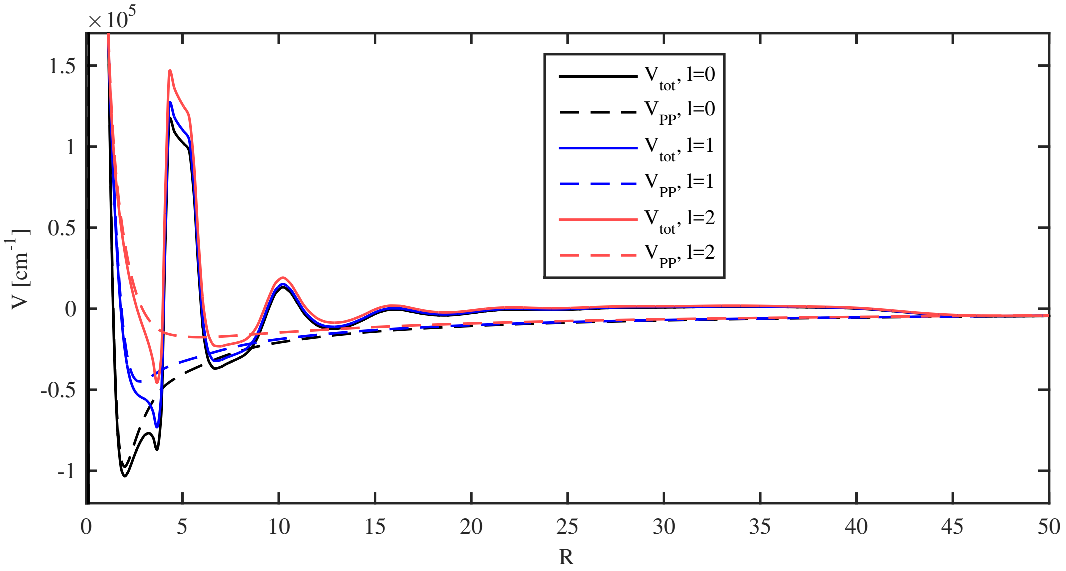

FIG. 7. The l-dependent total potential for the valence electron of a Na atom immersed into a helium droplet consisting of 1000 He atoms (solid line). The corresponding unperturbed pseudopotentials for Na are shown as dashed lines for comparison.

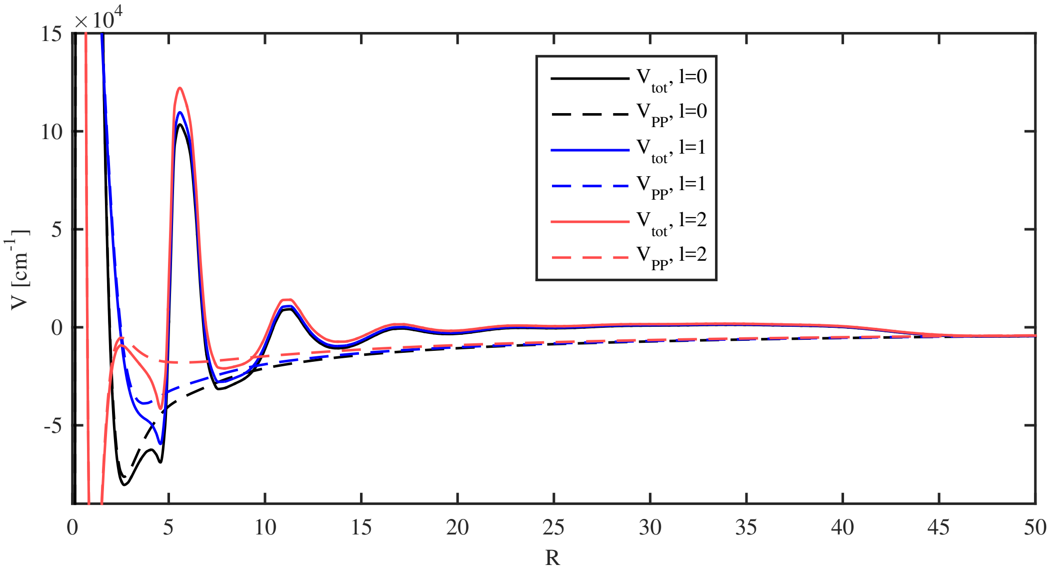

FIG. 8. The l-dependent total potential for the valence electron of a K atom immersed into a helium droplet consisting of 1000 He atoms (solid line). The corresponding unperturbed pseudopotentials for K are shown as dashed lines for comparison.

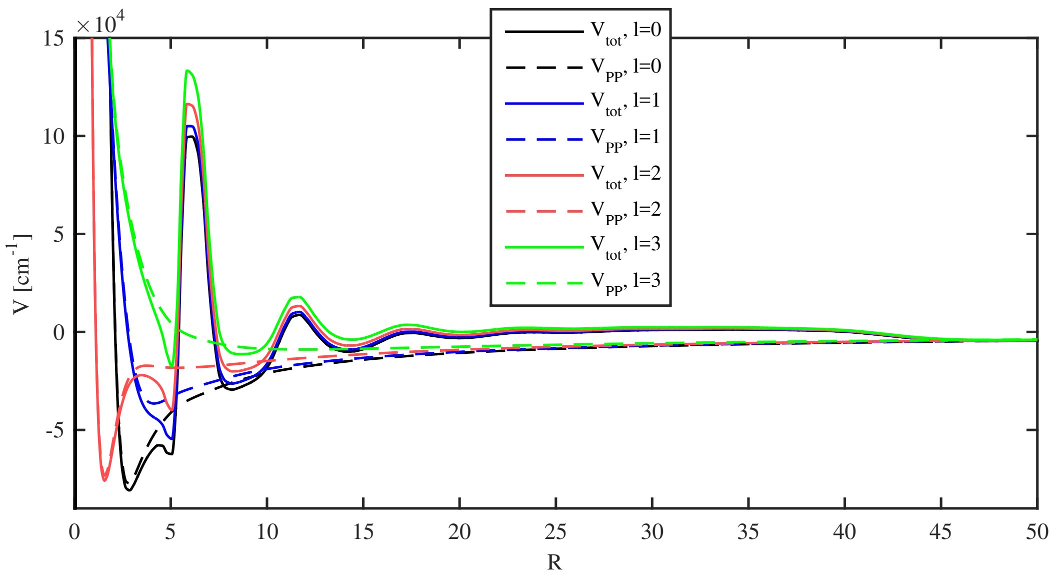

FIG. 9. The l-dependent total potential for the valence electron of a Rb atom immersed into a helium droplet consisting of 1000 He atoms (solid line). The corresponding unperturbed pseudopotentials for Rb are shown as dashed lines for comparison.

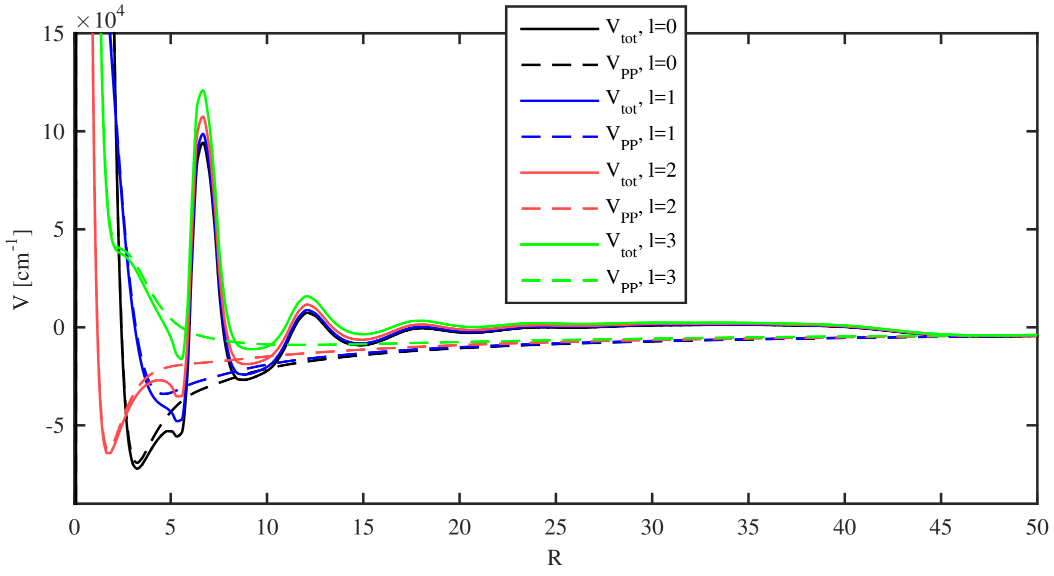

FIG. 10. The l-dependent total potential for the valence electron of a Cs atom immersed into a helium droplet consisting of 1000 He atoms (solid line). The corresponding unperturbed pseudopotentials for Cs are shown as dashed lines for comparison.

III. PROBABILITY DENSITIES

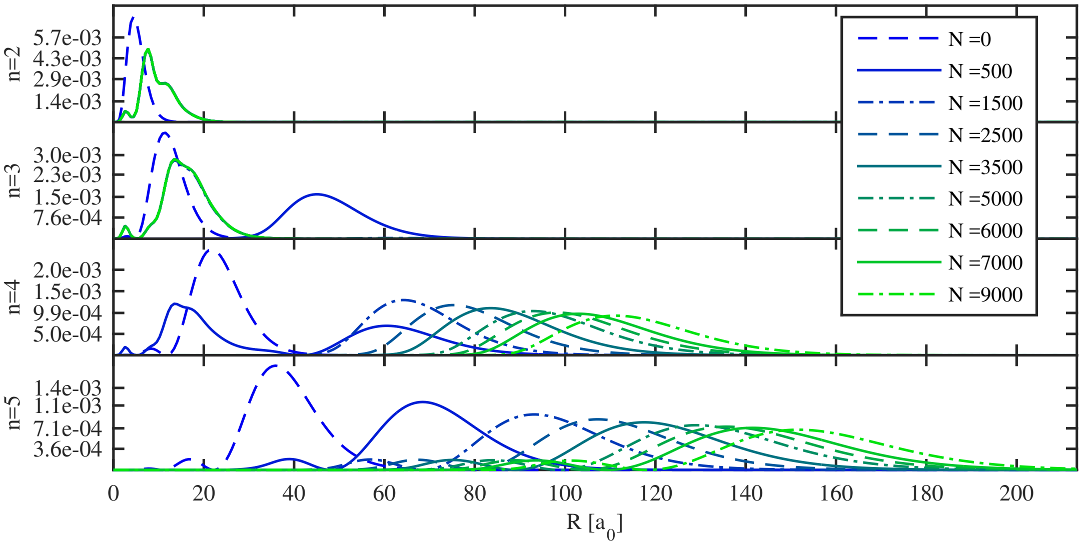

FIG. 11. The probability density of the Li valence electron is shown as a function of the radial distance for excited states with principal quantum numbers $n = 2-5$ and zero angular momentum. The unperturbed Li density ($N = 0$) is compared to the results obtained after immersion into He_N of increasing size.

The probability densities for a valence electron attached to an alkali ion enclosed in He are shown for Li, Na, K, and Cs in the figures 11, 12, 13, and 14 for different numbers of helium atoms (N). The corresponding figure for Rb is included in the main manuscript.The results are given for an orbital angular momentum of 0 and the lowest four principal quantum numbers. In this case we observe that the lowest two state are within the helium droplet, whereas states with larger n are continuously pushed outward.

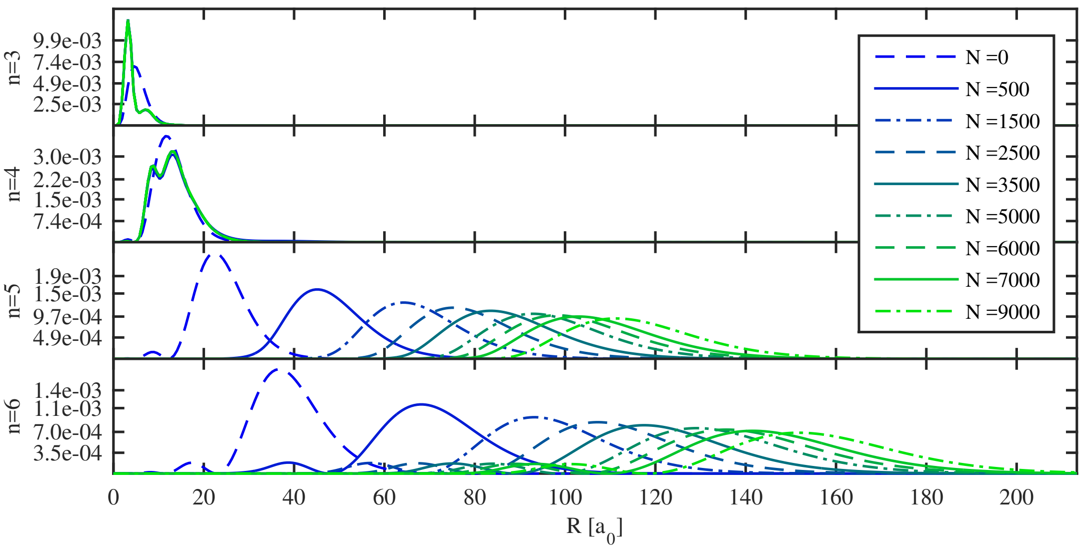

FIG. 12. The probability density of the Na valence electron is shown as a function of the radial distance for excited states with principal quantum numbers $n = 3-6$ and zero angular momentum. The unperturbed Na density ($N = 0$) is compared to the results obtained after immersion into He_N of increasing size.

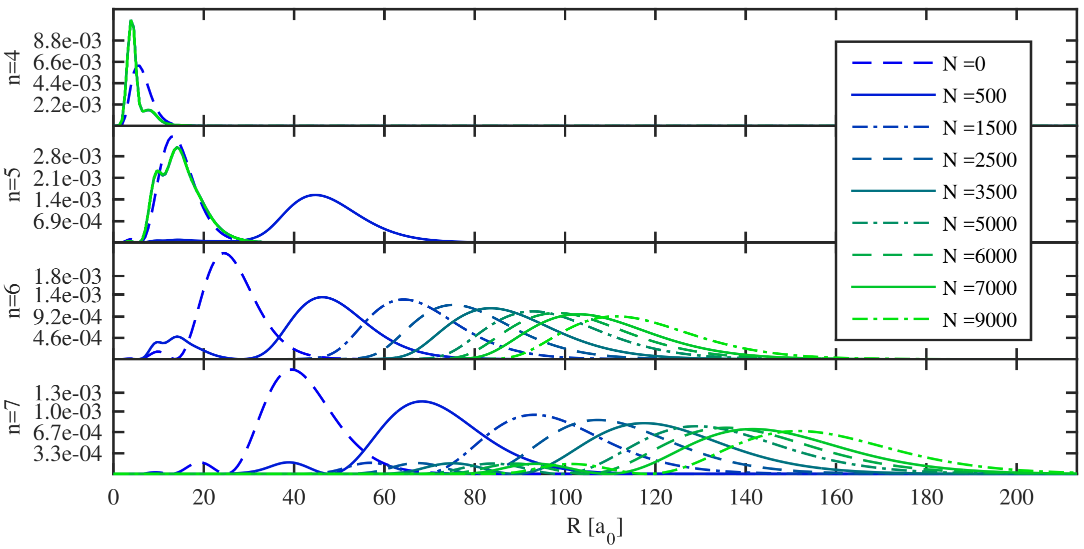

FIG. 13. The probability density of the K valence electron is shown as a function of the radial distance for excited states with principal quantum numbers $n = 4-7$ and zero angular momentum. The unperturbed K density ($N = 0$) is compared to the results obtained after immersion into He_N of increasing size.

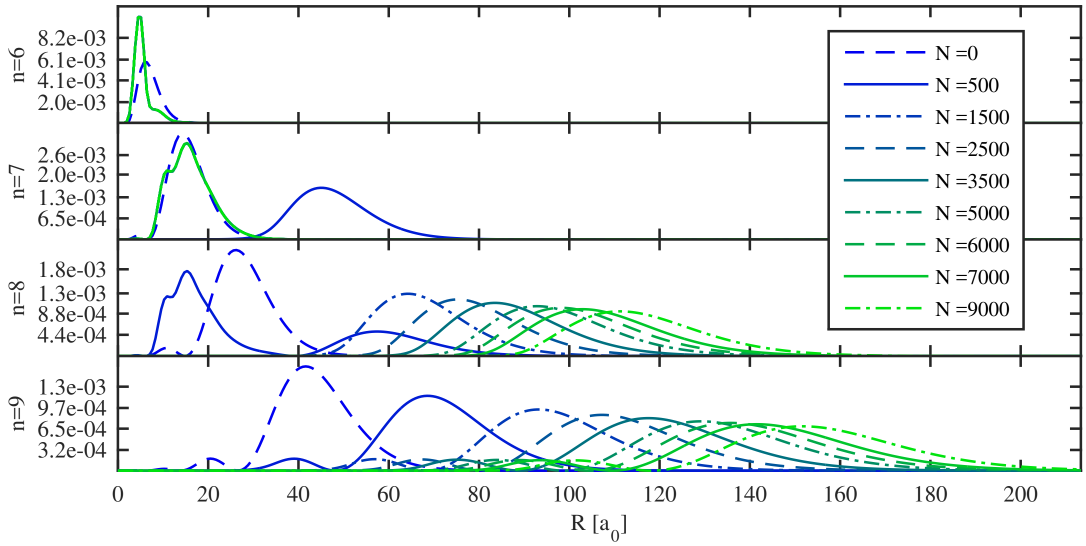

FIG. 14. The probability density of the Cs valence electron is shown as a function of the radial distance for excited states with principal quantum numbers $n = 6-9$ and zero angular momentum. The unperturbed Cs density ($N = 0$) is compared to the results obtained after immersion into He_N of increasing size.

IV. ENERGIES AND MEAN RADII FOR THE VALENCE ELECTRON IN THE COMBINED POTENTIAL

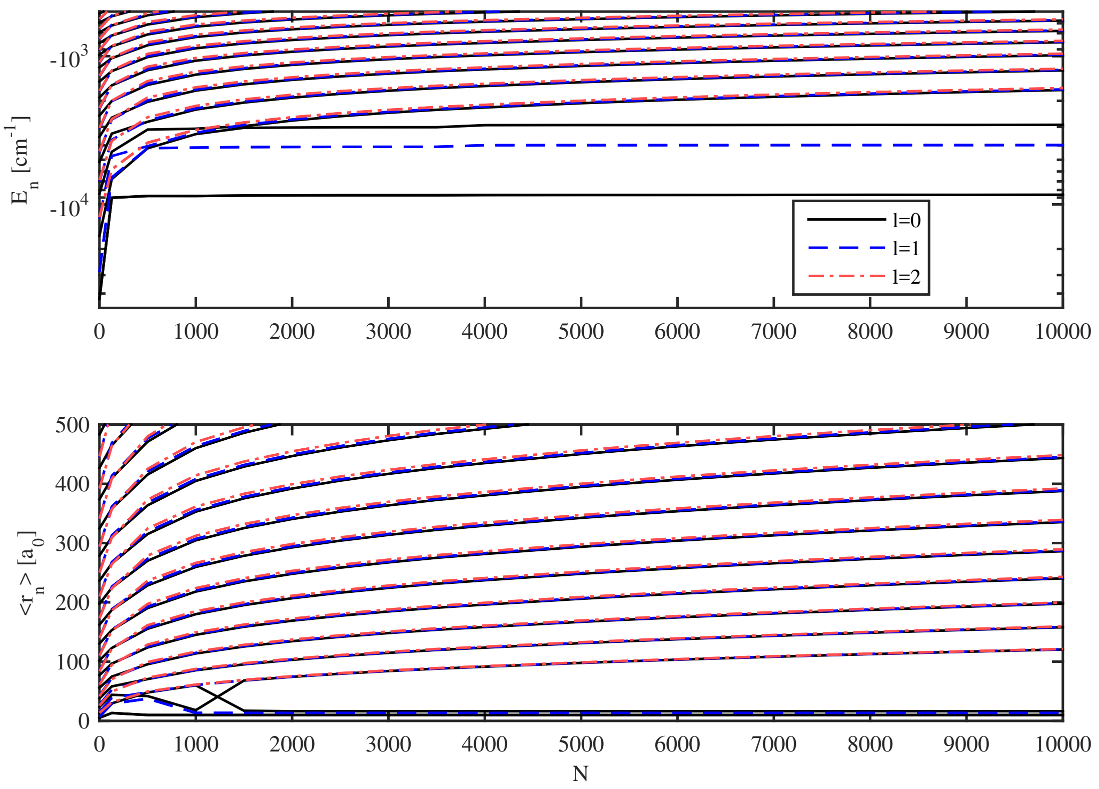

FIG. 15. The upper graphics shows the energies for excited states of Li with different principal quantum numbers depending on the He droplet size. Note that a logarithmic scale is used for the energy. The lower graphics shows their corresponding mean radii as a function of the He droplet size.

This part shows the eigenenergies and expectation values for the radius of the corresponding eigenstates of the valence electron in the combined potentials. The binding energy (upper part of the figures) decreases with N for all states. For the lowest states the energy becomes constant after a certain amount of helium is added, which indicates that these states lie within the droplet and are therefore not affected by the actual He droplet size. Also their radius remains constant. The other states show a decreasing binding energy for larger helium droplets. Their wave functions are continuously pushed outwards as can bee seen from the increasing radii in the lower graphics. The valence electron radii grow lin-

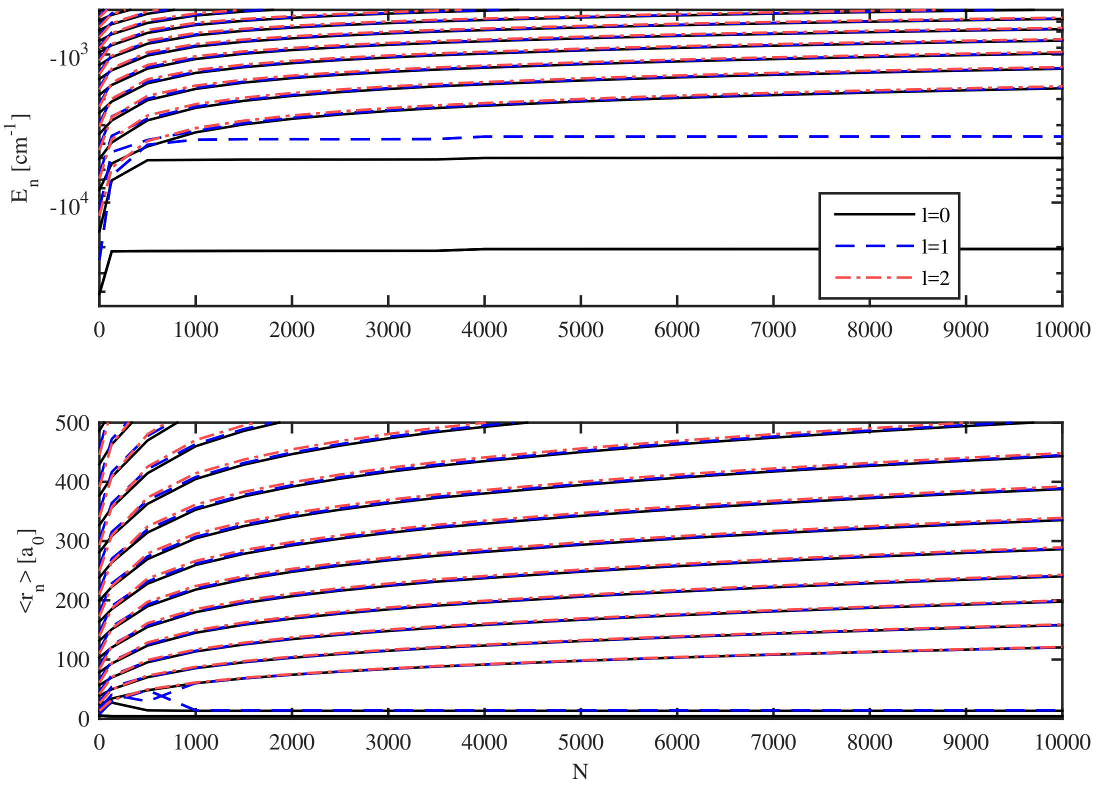

FIG. 16. The upper graphics shows the energies for excited states of Na with different principal quantum numbers depending on the He droplet size. Note that a logarithmic scale is used for the energy. The lower graphics shows their corresponding mean radii as a function of the He droplet size.

early with the droplet radius, which can be estimated from the number of He atoms by $r_{He} \approx 2.22 \cdot N^{1/3}\ \left[\text{Å}\right]$, see Ref. 5.

The jumps in the mean radius which appear in some cases are caused by energetically close eigenstates of the valence electron. One state is located inside the droplet close to the ionic core, the other one on the surface of the droplet, and minimal changes of the total potential can lead to a different ordering of the states.

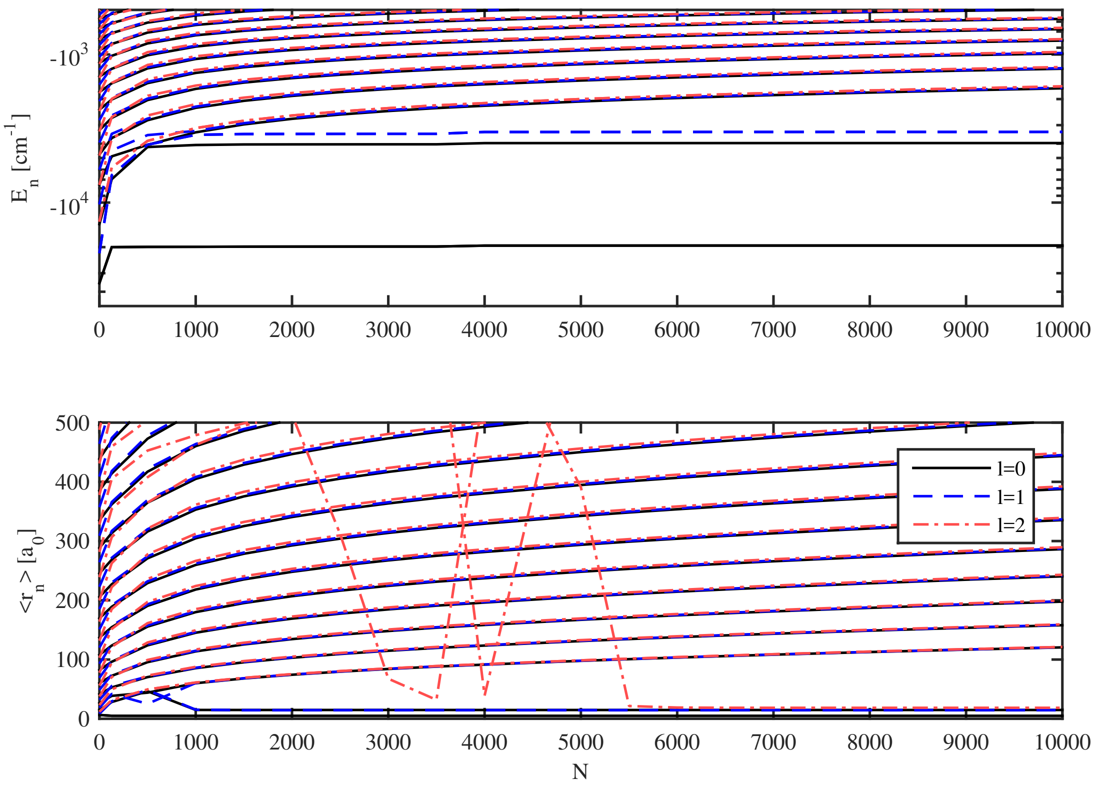

FIG. 17. The upper graphics shows the energies for excited states of K with different principal quantum numbers depending on the He droplet size. Note that a logarithmic scale is used for the energy. The lower graphics shows their corresponding mean radii as a function of the He droplet size.

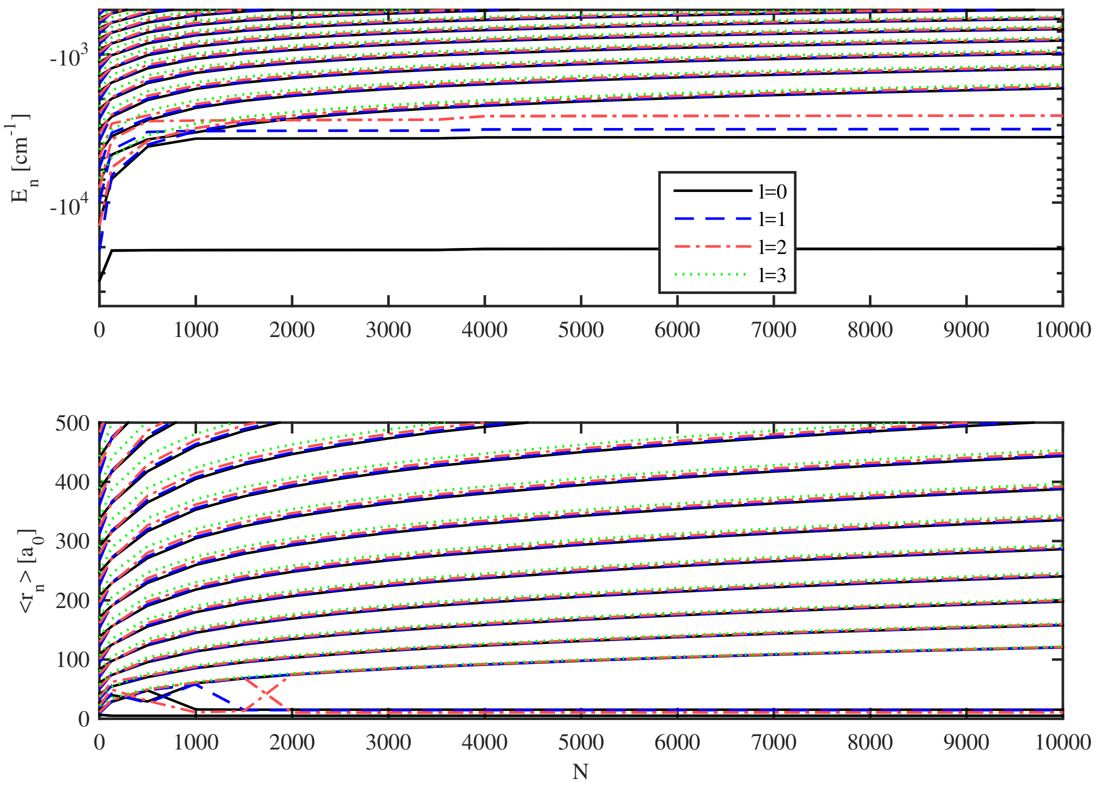

FIG. 18. The upper graphics shows the energies for excited states of Rb with different principal quantum numbers depending on the He droplet size. Note that a logarithmic scale is used for the energy. The lower graphics shows their corresponding mean radii as a function of the He droplet size.

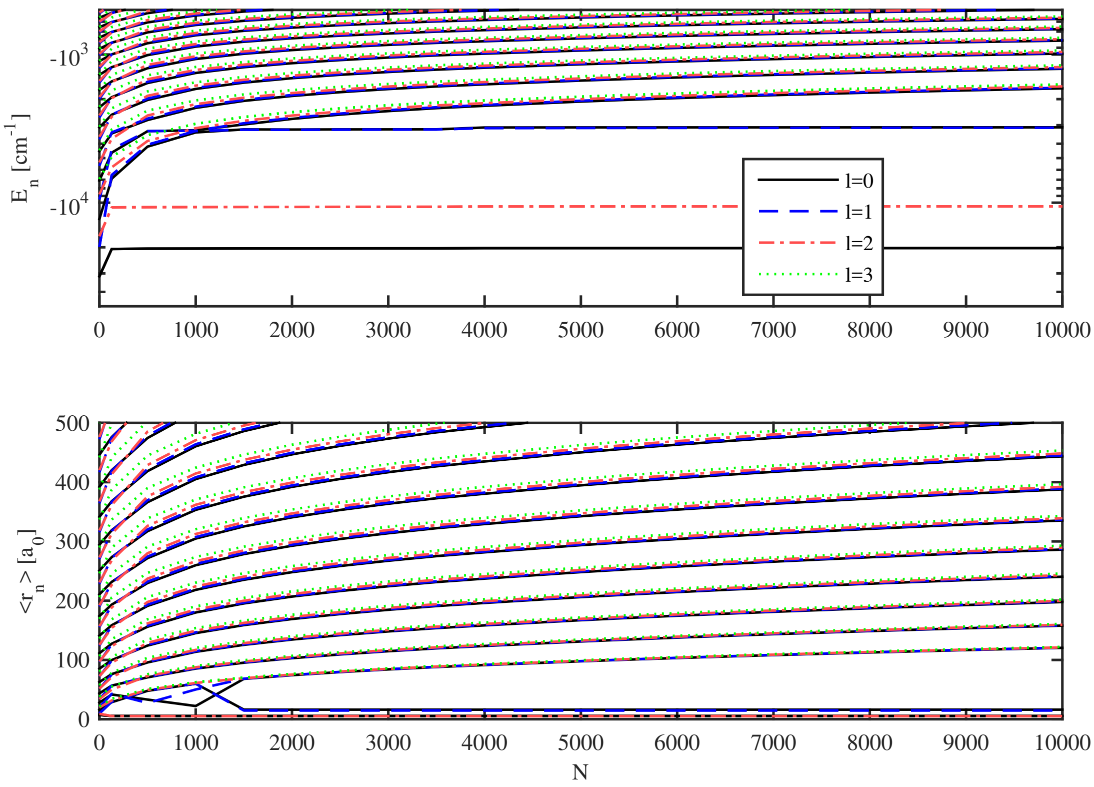

FIG. 19. The upper graphics shows the energies for excited states of Cs with different principal quantum numbers depending on the He droplet size. Note that a logarithmic scale is used for the energy. The lower graphics shows their corresponding mean radii as a function of the He droplet size.

V. INTERSECTION BETWEEN SOLVATION ENERGY AND BINDING ENERGY

This section analyzes the stability of the proposed systems where the ionic alkali metal core is fully immersed in the droplet while the valence electron is orbiting around the droplet. The results for all alkali metal atoms and different orbital angular momenta are presented in Figures 20 to 24. The system is stable if the electron is in an excited state with a principal quantum number above the lines depicted in the figures. In these states the energy costs due to the perturbation of the valence electron orbital are overcompensated by the energy gain through the immersion of the alkali metal ion. The break-even point depends slightly on the number of helium atoms as can be seen in Figures 20 to 24. These curves are obtained from the size-dependent intersection points of binding energy differences and solvation energies (see Fig. 8 of the manuscript e.g. for a comparison in He_{3000}).

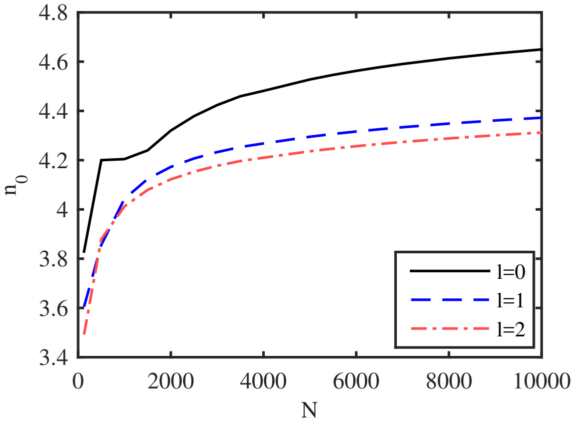

FIG. 20. The lowest principal quantum number of Li where energy costs due to the He perturbation can be compensated by immersion of the alkali metal ion, shown for different angular momenta, as a function of the He_N droplet size.

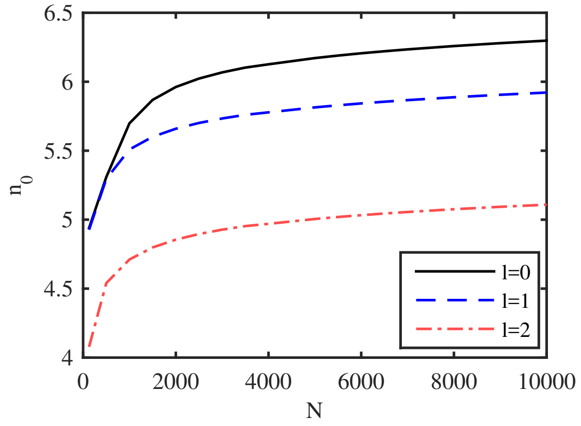

FIG. 21. The lowest principal quantum number of Na where energy costs due to the He perturbation can be compensated by immersion of the alkali metal ion, shown for different angular momenta, as a function of the He_N droplet size.

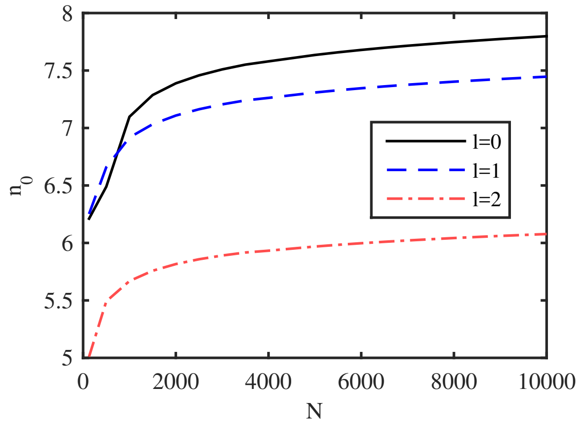

FIG. 22. The lowest principal quantum number of K where energy costs due to the He perturbation can be compensated by immersion of the alkali metal ion, shown for different angular momenta, as a function of the He_N droplet size.

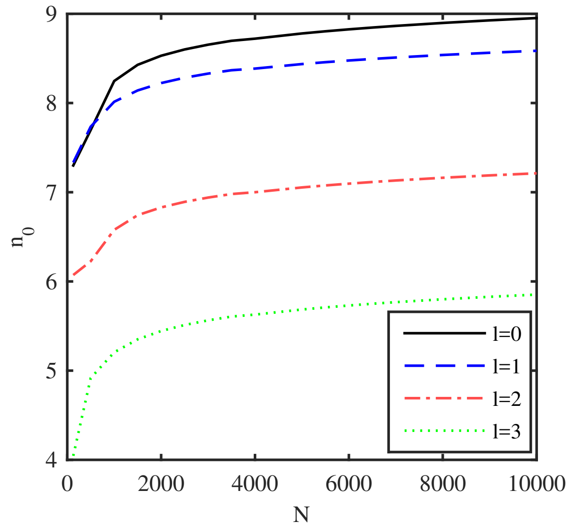

FIG. 23. The lowest principal quantum number of Rb where energy costs due to the He perturbation can be compensated by immersion of the alkali metal ion, shown for different angular momenta, as a function of the He_N droplet size.

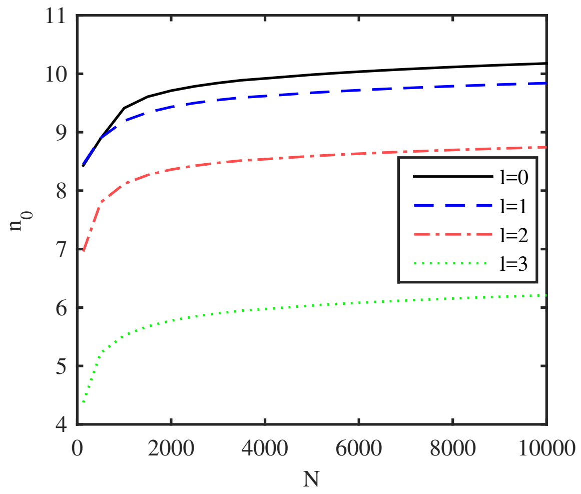

FIG. 24. The lowest principal quantum number of Cs where energy costs due to the He perturbation can be compensated by immersion of the alkali metal ion, shown for different angular momenta, as a function of the He_N droplet size.

[1] C. J. Lorenzen and K. Niemax, Phys. Scripta **27**, 300 (1983).

[2] Y. Ralchenko, A. Kramida, J. Reader, and NIST ASD Team, National Institute of Standards and Technology, Gaithersburg, MD (2015), available: http://physics.nist.gov/asd.

[3] E. Cheng, M. W. Cole, and M. H. Cohen, Phys. Rev. B **50**, 1136 (1994).

[4] E. Cheng, M. W. Cole, and M. H. Cohen, Phys. Rev. B **50**, 16134 (1994), erratum.

[5] F. Lackner, *Rydberg States of Alkali-Metal Atoms on Superfluid Helium Nanodroplets*, Doctoral Thesis, Graz Universtiy of Technology (2012).
